# Targeting Protein Aggregates with Natural Products: An Optional Strategy for Neurodegenerative Diseases

**DOI:** 10.3390/ijms241411275

**Published:** 2023-07-10

**Authors:** Lingzhi Xiang, Yanan Wang, Shenkui Liu, Beidong Liu, Xuejiao Jin, Xiuling Cao

**Affiliations:** 1 State Key Laboratory of Subtropical Silviculture, School of Forestry and Biotechnology, Zhejiang A&F University, Hangzhou 311300, China; xianglingzhi@stu.zafu.edu.cn (L.X.); wangyanan@stu.zafu.edu.cn (Y.W.); shenkuiliu@nefu.edu.cn (S.L.); beidong.liu@cmb.gu.se (B.L.); 2 Department of Chemistry and Molecular Biology, University of Gothenburg, 41390 Gothenburg, Sweden

**Keywords:** natural products, neurodegenerative diseases, protein aggregation, Aβ, tau, α-Syn, Htt, FUS

## Abstract

Protein aggregation is one of the hallmarks of aging and aging-related diseases, especially for the neurodegenerative diseases (NDs) such as Alzheimer’s disease (AD), Parkinson’s disease (PD), Huntington’s disease (HD), Amyotrophic lateral sclerosis (ALS), and others. In these diseases, many pathogenic proteins, such as amyloid-β, tau, α-Syn, Htt, and FUS, form aggregates that disrupt the normal physiological function of cells and lead to associated neuronal lesions. Protein aggregates in NDs are widely recognized as one of the important targets for the treatment of these diseases. Natural products, with their diverse biological activities and rich medical history, represent a great treasure trove for the development of therapeutic strategies to combat disease. A number of in vitro and in vivo studies have shown that natural products, by virtue of their complex molecular scaffolds that specifically bind to pathogenic proteins and their aggregates, can inhibit the formation of aggregates, disrupt the structure of aggregates and destabilize them, thereby alleviating conditions associated with NDs. Here, we systematically reviewed studies using natural products to improve disease-related symptoms by reducing or inhibiting the formation of five pathogenic protein aggregates associated with NDs. This information should provide valuable insights into new directions and ideas for the treatment of neurodegenerative diseases.

## 1. Introduction

Neurodegenerative diseases (NDs) are a heterogeneous group of disorders characterized by abnormal protein aggregation leading to the structural and functional degeneration of the central and peripheral nervous systems [1]. These diseases cause a large number of deaths and enormous medical costs worldwide, placing a heavy burden on patients, their families and society. According to the Global Alzheimer’s Disease Report, there are already more than 55 million people with Alzheimer’s disease (AD) worldwide, and this number is expected to rise to 78 million by 2030 and 152 million by 2050 [2]. Parkinson’s disease (PD) is the second most common and fastest growing ND in the world, with a global prevalence of more than 6 million people and a 2.5-fold increase from the previous generation of patients, and is expected to double again to more than 12 million by 2040 [3]. There is growing evidence that men are twice as likely as women to develop Parkinson’s disease, but women have higher mortality rates and a more rapid disease progression, placing an enormous burden on the population [4]. Huntington’s disease (HD) cases are distributed worldwide, with a prevalence of 4–10 per 100,000 in Western countries and approximately 5 per million in Asian populations [5,6]. The incidence of amyotrophic lateral sclerosis varies from country to country and region to region, from about (2–3/100,000) in Europe to about (0.7–0.8/100,000) in Asia, with huge annual treatment costs [7]. The common symptoms of these diseases are memory and cognitive impairment, as well as difficulties with speech and movement, and they tend to be more common in older people [8].

In these diseases, one or more different pathologically aggregation-prone polypeptides misfold and are packaged into large insoluble inclusion bodies. For example, the two hallmark pathological the features of AD are extracellular amyloid plaques composed of Aβ peptides and intracellular neurofibrillary tangles composed of hyperphosphorylated microtubule-associated protein tau [9,10]. PD is a movement disorder characterized by the accumulation of Lewy bodies in neurons, which are mainly composed of 𝛼-synuclein protein aggregates [11,12]. Due to CAG repeat expansion in the huntingtin (HTT) gene, the mutant Htt protein accumulates in neurons and forms deposits that produce cytotoxicity, leading to the development of HD [13]. And one of the reasons why ALS occurs is because the abnormally aggregated FUS protein state is more solidified, impairing its normal physiological function [14]. These diseases not only hinder people’s normal physical activities and increase their psychological stress, but also place a huge burden on society. However, there are no symptomatic drugs for these diseases. Therefore, finding ways to make the diseases more treatable has become a priority.

Natural products are a class of compounds isolated from plants or fungi that are biologically active and have a rich history of medicinal use [15]. In recent years, the biological activity, nutritional value, and potential health and therapeutic benefits of natural products have been intensively explored and studied [16,17]. Due to their neuroprotective effects, a variety of compounds from different sources have been proposed to have therapeutic efficacy in treating neurodegenerative diseases, and not only in alleviating their superficial symptoms [18,19,20]. Specifically, natural products can inhibit the formation of pathogenic protein aggregates and attenuate the neurotoxicity of pathogenic protein aggregates [16]. For example, natural products respond to autophagic pathways to reduce neurological damage such as oxidative stress from pathogenic protein aggregates [21,22,23]. In addition, natural products cleave β-amyloid structures to reduce aggregates formed by pathogenic proteins [24,25]. Similarly, natural products reduce levels of key enzyme activity that forms aggregates to inhibit oligomer formation [21,26,27]. Natural products offer new avenues for research into inhibiting the formation of disease-causing protein aggregates and thereby alleviating disease symptoms. Many scientists have carried out a lot of work in this direction, but there has not been a systematic review. Therefore, this review provides a systematic summary of natural products that affect pathogenic protein aggregates.

## 2. Study of Natural Products on Neurodegenerative Diseases

Throughout human history, natural products have been attractive alternatives for the prevention and treatment of disease, and have contributed to the development of modern medicines [28]. Natural products and their complex molecular frameworks provide a range of unknown chemical species for medicinal chemists discovering chemical probes and drugs [29]. Natural products have long provided a valuable source for exploring drugs to treat disease [29]. In recent years some natural products, such as honey, ginseng extract, resveratrol (RES), curcumin, epigallocatechin gallate (EGCG), etc., have attracted much attention for the treatment of neurodegenerative diseases due to their anti-inflammatory and antioxidant properties. Honey has been reported to reduce oxidative stress in the brain and improve morphological damage in the hippocampus and medial prefrontal cortex, and morphological damage in the prefrontal cortex [30]. In the 1-methyl-4-phenylpyridiniumion (MPP+)-induced apoptosis in rat pheochromocytoma (PC12) cells, Korean red ginseng inhibits apoptosis and prevents the reduction in cell survival by decreasing caspase-3 and caspase-9 mRNA expression [31]. Resveratrol significantly inhibited Aβ-induced proliferation and activation of BV-2 cells, as well as the release of their pro-inflammatory cytokines, IL-6 and TNF-a, in a dose-dependent manner (10–50 nM). It also attenuates neuroinflammation by inhibiting the TXNIP/TRX/NLRP3 signaling pathway [32]. Many studies have shown that Resveratrol not only reduces neuroinflammation, but also reduces oxidative-stress-induced neurological damage through AMP-activated protein kinase (AMPK) and SIRT1 [33,34,35,36]. EGCG can promote the ROS reaction by chelating its phenolic groups with other metal ions, leading to a decrease in the amount of the free form of the metal [37]. In addition, the antioxidant capacity of EGCG is also based on increasing the activity of glutathione peroxidase and superoxide dismutase [38]. 

The current summary of studies on natural product protection of the nervous system against neurodegenerative diseases focuses on the role of natural products in antioxidant, neuroinflammatory, mitochondrial dysfunction, and apoptosis events. Sairazi et al. [16] summarized that some natural products reduce the pathological features of neurodegenerative diseases through antioxidant and anti-inflammatory mechanisms of action. Andrade et al. [39] described the efficacy of natural products in the clinical treatment of neurodegenerative diseases. According to the summary of previous experiments, it is known that to investigate the effect of natural compounds on Aβ, tau protein, and brain volume loss, a total of 119 volunteers were given 500 mg of oral resveratrol for 52 weeks, but there was no significant reduction in the levels of biomarkers of neurodegenerative diseases such as Alzheimer’s [40]. A great deal of work has been carried out on the effect of compounds on NDs. For example, curcumin is rich in antioxidant, anti-aging, anti-inflammatory, and anti-diabetic bioactivities, among others [41,42,43]. In previous studies, the oral administration of curcumin attenuated memory deficits in AD mice and alleviated inflammation by inhibiting the HMGB1-RAGE/TLR4-NF-κB signaling pathway in amyloid precursor protein/presenilin 1 (APP/PS1) transgenic mice AD model [44]. Curcumin reduces β-amyloid-induced neurological damage by up-regulating type 2 superoxide dismutase (SOD2) expression in HT22 cells [45]. However, there are few systematic summaries of natural products that directly target pathogenic protein aggregates in neurodegenerative diseases. Therefore, we summarize natural products that directly target aggregates, as shown in Table 1. Natural products inhibit the formation of pathogenic protein aggregates or disassemble their structure, and attenuate the neurotoxicity caused by aggregates.

## 3. Natural Products Reduce Amyloid-β Aggregates and Toxicity

### 3.1. The Process of Amyloid-β Protein Formation

**Figure 1:**
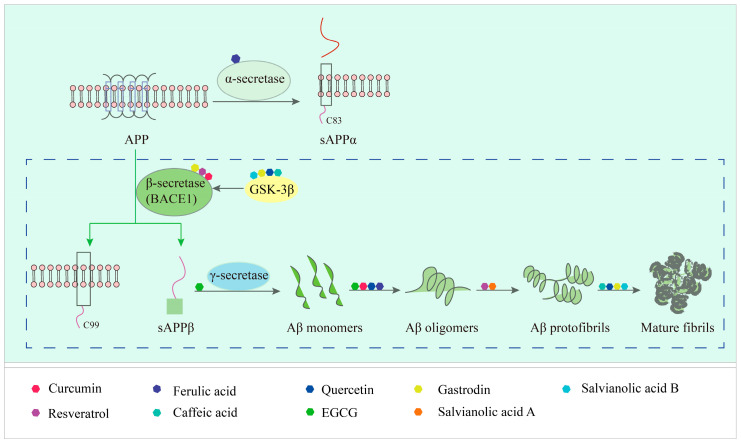
Natural products targeting the Aβ formation phase: inhibiting amyloid production. Caffeic acid (CA), gastrodin, quercetin, and salvianolic acid B inhibit the formation of Aβ aggregates by attenuating glycogen synthase kinase (GSK3β) enzyme activity and reducing BACE1 (β-secretase) activity. Curcumin and resveratrol (RES) reduce the level of BACE1 expression, thereby decreasing oligomer formation. Epigallocatechin-3-gallocatechin (EGCG) directly binds to oligomers and remodels the structure of oligomers, thereby reducing Aβ-induced neurotoxicity. EGCG specifically binds directly to oligomers and remodels their structure, disrupting their structure and attenuating Aβ-induced neurotoxicity.

Amyloid-β (Aβ) aggregation is one of the key pathologies in AD [87]. The neurotoxicity caused by Aβ deposition produces various destructive stimuli in the central nervous system, triggering a series of pathologies such as synaptic degeneration, tau hyperphosphorylation, oxidative stress, neuroinflammation, neuronal degeneration, and neuronal deficits. The precursor protein of Aβ (APP) is a protein of 38–43 amino acid residues that can be cleaved by three types of secretases: α-secretase (ADAM10), β-secretase (BACE1), and γ-secretase (PS1) [88]. Under normal conditions, APP is cleaved by α-secretase (extracellular structure) into a fragment consisting of 83 amino acids (C83) and an extracellular structural domain (sAPPα). sAppα is further cleaved by γ-secretase. The cleavage site of α-secretase prevents the production of Aβ, which facilitates neuronal protection and cellular value creation. In pathological cases, APP is cleaved by β-secretase (extracellular structure) to form a C-terminal membrane-forming fragment (C99) and an extracellular structural domain (sAPPβ), and sAPPβ is further cleaved by γ-secretase to form Aβ [88]. There are two main forms of Aβ in APP: one with 40 amino acids (Aβ40), which is more fibrillogenic, and one with 42 amino acids (Aβ42) [89]. Aβ exists in an aqueous solution as a mixture of α-helix and β-sheet, and the β-sheet leads to Aβ aggregation, thus triggering Aβ neurotoxicity. Therefore, inhibition of the β-sheet plays an important role in inhibiting Aβ aggregation. A number of studies have been conducted to find methods that can inhibit Aβ aggregation based on pathological findings caused by its aggregation [90,91]. Small molecule natural products have been found to be effective in inhibiting the aggregation of Aβ oligomers, protofibrils, and fibrosis, thus significantly reducing the harmful toxicity caused by Aβ deposition. As shown in Figure 1, different structural features of different natural products result in different mechanisms of action which inhibit Aβ aggregation.

### 3.2. Specific Description of Natural Products Targeting Amyloid-β Action

**Curcumin.** The unique molecular structure of curcumin plays a pivotal role in its pharmacological effects [92]. As observed via atomic force microscopy (AFM) and transmission electron microscopy (TEM), curcumin can specifically bind directly to the N-terminal of an Aβ monomer (residues 5–20), which is covered within Aβ oligomers at 1–2 nm to strongly inhibit the formation of Aβ deposits [46]. Curcumin deformed the β-sheet structure through hydrophobic interactions and hydrogen bonding in the molecular structure. In addition, π-stacking between curcumin and the aromatic residues of Aβ led to the reduction of the β-sheet structure. Curcumin reduced the β-sheet content in Aβ without affecting the monomer contact, as studied by the all-atom explicit solvent molecular dynamics simulation method [47]. Curcumin attenuated Aβ-membrane interactions in Aβ-40 (1–40 μM), induced injury in SH-SY5Y cells in a dose-dependent manner (0–5 μM), ameliorated Aβ-induced neurotoxicity, and reduced the rate and extent of Aβ insertion into the anionic lipid monomolecular layer [48]. In the *Drosophila* model of AD, curcumin promoted the conversion of amyloidogenic fibers by reducing the pre-fiber/oligomeric species of antibodies, thereby reducing neurotoxicity in *Drosophila* [49]. In a scopolamine-induced AD mice model, curcumin reduced the formation of Aβ aggregates by downregulating glycogen synthase kinase (GSK3β) enzyme activity, an enzyme known to regulate BACE1 activity [93]. In SH-SY5Y cells, curcumin inhibits the transcriptional and translational levels of BACE1 enzymes by selectively activating estrogen receptor β (ERβ), which directly affects the nuclear factor kappa B (NFκB) signaling pathway [94]. However, curcumin is insoluble in water, and more and more studies are being conducted on the development of curcumin analogues or derivatives in order to better exploit the role of Curcumin in anti-AD [50]. Analogs of curcumin not only inhibit the formation of Aβ aggregates as well as curcumin, but are also more than 160 times more water soluble than curcumin. A curcumin analog (CLC-R17) has significantly reduced Aβ deposition in a mouse model of AD. In an animal cell model of AD, CLC-R17 effectively reduced the levels of Aβ in conditioned media and decreased the levels of oligomeric amyloid in cells. CLC-R17 attenuated the maturation of amyloid protein precursors in the secretory pathway, upregulated PS1 enzyme activity, decreased BACE1 enzyme activity, and attenuated Aβ-induced neurotoxicity [95]. Molecular dynamics simulations indicates that curcumin derivatives can partially dissociate the outermost peptide of Aβ(1–42) protofibrils by disrupting the β-sheet structure [96]. Curcumin can also be combined with nanomaterials to form novel multifunctional nanomaterials that can significantly reduce the β-amyloid plaque burden in APP/PS1 transgenic mice, reduce oxidative stress damage from Aβ deposition, and successfully rescue memory deficits in mice [97,98,99].

**Resveratrol (RES).** In SAMP8 mice, an animal model of aging and AD, RES has protected against APP processing into Aβ amyloid by reducing BACE1 and APP gene expression [54]. RES plays an important role in promoting the cleavage of non-amyloid proteins from amyloid precursor proteins [55]. Through thioflavin (ThT) and matrix-assisted laser desorption ionization time of flight (MALDI-TOF) mass spectrometry, RES cleaved Aβ(1–42) peptides into shorter fragments, Aβ(1–42) oligomeric protofibrillated and fibrillated structures which cannot be observed via AFM, and reduced the height of Aβ(1–42) aggregates (0.675–3.275 nm) [56]. In the SH-SY5Y cell line induced by the Aβ(1–42) peptide (10 μM), RES (1 μM) reduced Aβ(1–42)-induced toxicity (from 100% to 78%) and restored cell viability [56]. RES enhances the clearance of amyloid peptides and reduces neuronal damage. For example, RES improved cognition and amyloid plaque formation by reducing Aβ deposition and significantly reducing BACE1 enzyme levels in Tg6799 mice, a transgenic mouse model with five familial AD mutations [57].

**Ferulic acid (FA).** FA is mostly present in the cell wall as a trans-isomer and esterified with a variety of specific polysaccharides, giving the cell wall a stable and rigid structure [100]. In a previous in vitro study, FA (50 μM) was found to inhibit the formation and elongation of β-amyloid (fAβ (1–40) and fAβ (1–42)) in a dose-dependent manner, observed at 37 °C and a pH of 7.5 using fluorescence spectroscopy ThT and electron microscopy (IC50 of 5.5 µM) [60]. Among the Aβ peptide structures, α-helix and parallel β-turn are the major structures of individual Aβ peptides, followed by antiparallel β-turns. It is known from 1 µs molecular dynamics (MD) simulation experiments in the presence of FA, the tendency of α-helices increases, parallel β-turns decreases, and antiparallel β-turns almost disappears, which proves that FA increases the tendency of Aβ helices, decreases the tendency of non-helical Aβ peptides, and prevents the formation of dense body nuclei [61]. In the *Caenorhabditis elegans* model, FA significantly suppressed Aβ-induced paralysis and pathological symptoms of hypersensitivity to exogenous serotonin by activating the HLH-30 transcription factor to nuclear localization, reducing lipid levels upstream of autophagy, while increasing the expression of the autophagy reporter gene LGG-1. It also reduced Aβ monomers, oligomers, and deposits by 50–70% in a dose-dependent manner (100 μM), effectively reducing Aβ-induced neurotoxicity [100]. Recent studies have shown that endothelin-1 (ET1)-mediated action on the ET1 receptor (ETRA) triggers the constriction of brain capillaries, which may exacerbate Aβ deposition. In APP/PS1 transgenic mice, FA inhibited ETRA to counteract ET1-mediated constriction of brain capillaries, resulting in reduced hippocampal capillary density and diameter, attenuated Aβ aggregation, and spatial memory deficits [101].

**Caffeic acid (CA).** In the Aβ(25–35)-induced AD mouse model, CA attenuated Aβ-induced oxidative stress and neurotoxicity by reducing lipid peroxidation and nitric oxide (NO) production in the brain [102]. In Aβ(25–35)-induced PC12 cells, CA reduced GSK3β enzyme activity and intracellular calcium flux in a dose-dependent manner (4, 20 and 100 μg/mL), and protected against Aβ-induced neurotoxicity. CA, specifically bound to the amyloid C-terminal peptide, exhibited potent inhibitory activity against Aβ(1–42) fibrogenesis, scavenged Aβ(1–42)-induced oxidative stress, and inhibited Aβ(1–42)-induced neurotoxicity at a semi-inhibitory concentration of 4 μM in SH-5Y5Y cells [62]. In addition, CA (300 μM) prolonged the mean lifespan of *Caenorhabditis elegans* by 15.57% and protected against Aβ neurotoxicity by activating the transcription factor DAF-16 and its downstream targets SOD-3 and GST-4 [103]. In the course of the research, to promote the chemical stability of CA, transferrin (Tf)-loaded nanoparticles (NPs) were delivered across the blood–brain barrier (BBB) by coupling Tf to the surface of liposomes, taking advantage of the overexpression of Tf receptors in brain endothelial cells. Caffeic-acid-loaded Tf-functionalized liposomes prevented the aggregation of Aβ and the formation of protofibrils, and broke down the mature protofibrils, allowing for the efficient utilization of CA [104].

**Quercetin.** In vitro and silico structural studies showed that Quercetin inhibited BACE1 enzyme activity through hydrogen bond formation, with the hydroxyl group at the C3 position playing an important role [105,106]. By measuring the fluorescence of the single tyrosine intrinsic fluorophore (Tyr) of Aβ, quercetin was found to bind in a dose-dependent manner (50 μM) to β-amyloid oligomers at an early stage of aggregation, leading to the formation of modified oligomers that hinder the formation of neurotoxic β-sheet structures [63]. Furthermore, in hippocampal neurons with Aβ (5 μM)-induced injury, quercetin (10 μM) reduced oxidative stress injury, decreased ROS production, restored normal mitochondrial morphology, and prevented a decrease in mitochondrial membrane potential (50%) [64]. In vivo, quercetin extended the lifespan of AD *Drosophila*, rescued impaired climbing ability, and suppressed Aβ-induced neurotoxicity by restoring the expression levels of the cell cycle protein cyclin B [107]. Quercetin also protected human brain microvascular endothelial cells from toxicity of fibrillar Aβ1–40 (fAβ1–40) (20 μmol/L) in a dose-dependent manner (0.3–30 μmol/L), increased cell viability, reduced intracellular ROS production to protect cells, and significantly restored the expression levels of enzymes involved in the brain microvascular barrier generation (γ-GT and ALP) [108].

**Epigallocatechin-3-gallocatechin (EGCG).** EGCG inhibits amyloid aggregation mainly through three general mechanisms: the first is to directly bind to oligomers to disrupt their structure [66]. The second is to remodel oligomers and change their structure [67,68,69]. The third is to chelate with metal ions to inhibit their toxicity [70,71,72]. EGCG recognizes unfolded peptides and directly binds to the backbone of all proteins, stimulating the formation of non-toxic, non-pathway oligomers and reducing the toxicity of Aβ42 by approximately 40% [66]. EGCG reconstructed the Aβ structure following the Hill–Scatchard model, and the Aβ(1–40) self-association can occur cooperatively, generating Aβ(1–40) oligomers with multiple independent binding sites for EGCG, with a Kd ∼10-fold lower than that of the Aβ(1–40) monomers [68]. The solvent exposure of Aβ(1–40) oligomers was reduced upon binding to EGCG, while the β region involved in the direct monomer–fibril contact was remodeled in the absence of EGCG. EGCG has the ability to remodel large mature Aβ aggregates into small, amorphous aggregates that are not toxic to cells [69]. Metal ions such as Cu(II), Zn(II), and Fe(II) promote the fibrillation of the Aβ protein [71], while EGCG effectively disrupts the metal-induced Aβ aggregate formation pathway and reduces metal-induced Aβ aggregate neurotoxicity [72]. Using multiple all-atom molecular dynamics simulations, EGCG disrupted Aβ aggregation by the cell membrane on Aβ42 protofibrils in the presence of mixed POPC/POPG (7:3) lipid bilayers. EGCG tended to bind to the cell membrane and this binding altered the binding pattern between Aβ42 protofibrils and lipid bilayers, resulting in thinner and fewer membranes. EGCG played an important role in protecting cell membranes and attenuating Aβ toxicity [109].

**Gastrodin.** Gastrodin inhibited the aggregation of Aβ42 and promoted Aβ clearance, prevented Aβ42-induced neurotoxicity in SH-SY5Y cells, reduced the levels of Aβ plaques and hyperphosphorylated tau, and attenuated glial cell activation and pro-inflammation. Gastrodin inhibited the Aβ aggregation by decreasing the expression levels of Aβ transport enzymes (sAPPα, BACE1, RAGE), and prevented the activity of GSK3β, thereby reducing its neurotoxicity [76]. Gastrodin improved cognitive impairment in mice by increasing the levels of superoxide dismutase (33%) and glutathione peroxidase (39%), and reducing the levels of malondialdehyde (33%), the end product of lipid peroxidation in the brains of APP/PS1 transgenic mice [76]. In NPCs (neural progenitor cells), gastrodin (50 μg/mL) not only attenuated Aβ-induced neurotoxicity by reversing the Aβ(1–42)-induced increase in phosphorylation of MEK-1/2, extracellular-signal-regulated kinase (ERK) and c-JunN-terminal kinase (JNK), but also inhibited Aβ-induced neurotoxicity by reducing the production of pro-inflammatory factors TNF-α, IL-1β, IL-6, and NO (a potent inflammatory mediator) [110,111]. Furthermore, in Aβ(1–42)-injected C57BL/6 mice, gastrodin improved hippocampal neurogenesis by increasing the number of SOX-2 and double corticotropin (DCX)-positive cells (neural progenitor cells and differentiated neurons) in the DG (dentate gyrus) region. In vivo, Gastrodin inhibited Aβ deposition and improved memory deficits in mice by attenuating the activation of microglia and astrocytes in Tg2576 mice (a mice model of AD) [112]. In vitro, Gastrodin significantly altered the SOD and CAT activity levels and upregulated nuclear factor E2-related factor 2 (Nrf2) gene expression and extracellular signal-regulated kinase 1 and 2 (ERK1/2) phosphorylation to ameliorate Aβ(1–42)-induced neurotoxicity in primary cultured rat hippocampal neurons [113].

**Salvianolic acid A and salvianolic acid B.** Salvianolic acid A stabilized the β-sheet structure and inhibited Aβ42 aggregation in a dose-dependent manner, with an optimal effect at 40 µM and a semi-inhibitory concentration of 1–4 µM, and also broke down Aβ42 aggregation for protofibrillary aging in a dose-dependent manner, with an optimal concentration of 50 µM. In the SH-5Y5Y cell line, salvianolic acid A reduced oxidative stress damage in a dose-dependent manner (6.25–100 µM) and increased cell viability in Aβ-induced cytotoxicity [114]. In vitro, in the SH-SY5Y cell line overexpressing the human APP Swedish mutant (APPsw) model, salvianolic acid B increased the expression level of ADAM10 and decreased the expression level of BACE1 by increasing the activity of SOD and GSH-Px, and inhibiting the activity of GSK3β in a dose-dependent manner (0–100 μM), thereby reducing the expression levels of Aβ40 and Aβ42 and inhibiting the formation of Aβ aggregates [74]. Through thioflavin T fluorimetry (ThT) and an Aβ aggregating immunoassay (ELISA), salvianolic acid B was found to inhibit Aβ40 fibril aggregation (IC50: 1.54–5.37 mM) and destabilize preformed Aβ40 fibrils (IC50: 5.00–5.19 mM) in a dose-dependent (1–100 μM) and time-dependent (3–7 d) manner [115]. Salvianolic acid B not only protected cell viability by reversing the expression of BPRP protein (brain–pancreatic relative protein A expression) and reducing ROS and intracellular calcium production in Aβ(25–35)-induced PC12 cells, but also reduced Aβ aggregation by reducing Aβ fibrillation, thereby improving cell viability [116]. Moreover, in vivo, salvianolic acid B significantly attenuated glutathione (GSH) and lipid oxidation in neurons, and inhibited mitochondrial superoxide overproduction in Aβ-attacked neurons. At the same time, salvianolic acid B exhibited a strong protective function on mitochondrial bioenergetics and counteracted Aβ toxicity by preserving mitochondrial membrane potential and ATP production, as well as rescuing the enzymatic activities of cytochrome C oxidase and F1Fo ATP synthase in primary cultured mice neurons [117]. In addition, salvianolic acid B can inhibit Aβ formation by reducing the expression of BACE1 and increasing the expression of ADAM10 in Porphyromonas-gingivalis-infected mice [75,118].

In addition to these, there are other natural products that may play a role in inhibiting the formation of Aβ aggregates. HX106N is a botanical blend extract of *Dimocarpus longan*, *Liriope platyphylla*, *Salvia miltiorrhiza*, and *Gastrodia elata*. HX106N blocked Aβ aggregation at early pathological stages in a dose-dependent manner by binding to Aβ monomers and preventing their conversion to oligomers and fibrils. The blockade of mature β-sheet structures by salvianolic acid A, B, E, and rosmarinic acid in HX106N highlighted the inhibitory activity on Aβ aggregation [119]. In vivo, iso-orientin (6-C-glycosylflavone) treatment of amyloid precursor protein/presenilin 1 (APP/PS1) transgenic mice improved the viability of microglia in the mouse brain and reduced the expression levels of pro-inflammatory factors TNF-α, IL-6, and IL-1β, as well as cyclooxygenase-2 (COX-2), a key enzyme in microglial activation. At higher concentrations, iso-orientin reduced GSK3β expression levels, Aβ42 levels, and Aβ deposition, and improved the learning ability and memory in APP/PS1 transgenic mice. In vitro, iso-orientin (50 μM) reduced GSK3β activity and attenuated Aβ42 (5 μM)-induced neurotoxicity via the NF-KB pathway in SH-SY5Y cells [77,120]. In an in vitro co-culture model of mature neurons and neuronal cells, apigenin (4,5,7-trihydroxyflavone) (1 µM) rescued the morphology of neuronal cells exposed to Aβ oligomers (500 nM) for 4 h and modulated Aβ toxicity through anti-inflammation [121]. In vivo, apigenin restored mitochondrial dysfunction by significantly interfering with cytochrome c release and caspase 9 activation, with protective effects on working memory and neurology [122]. In a transgenic *Drosophila* AD model, apigenin reduced the formation of Aβ aggregates in a dose- (25, 50, 75 and 100 μM) and time-dependent manner (30 d). The formation of Aβ42 aggregates was significantly reduced 1.35-, 1.52-, 1.91-, and 2.39-fold, respectively, compared to the experimental group not treated with apigenin. Meanwhile, apigenin delayed the impaired climbing ability of *Drosophila* AD 1.34-, 1.61-, 2.23-, and 2.67-fold, respectively, compared to the control group [123]. In vitro studies of luciferase deficiency analysis revealed that cosmosiin (1, 5, 10 µM) (apigenin 7-O-β-glucoside), a derivative of apigenin, enhanced the first 144 nucleotides of the 5’UTR translation, thereby increasing the expression of ADAM10 and significantly reducing the levels of Aβ(1–40) and Aβ(1–42) in SH-SY5Y or HEK293 human cell lines [124]. In N2a/SweAPP cell lines, macelignan, a natural compound extracted from *Myristica fragrans*, dose-dependently (0, 5, 10, 15, and 20 µM) reduced BACE1 enzyme translation levels and APP protein expression through the PERK/eIF2α pathway to attenuate Aβ deposition [125]. Rosmarinic acid is a polyphenolic compound isolated from the rosemary plant of the Labiatae family, with a variety of physiological properties such as antioxidant, anti-inflammatory, antibacterial, antidepressant, and wound healing properties [126]. In a recent study, rosmarinic acid (0.25 mg/kg/day) significantly improved cognitive impairment and Aβ(25–35)-induced oxidative damage in mice after a 14-day administration [127]. Rosmarinic acid significantly improved spatial and recognition memory deficits induced by Aβ(1–42) in mice, and normalized neuronal density and the expression of neurogenic, synaptic markers [128]. In vitro and vivo experiments demonstrated that rosmarinic acid inhibits the formation of Aβ aggregates, disrupts the deposition of Aβ oligomers, and directly binds to Aβ tangles (EC50 = 20.3 µM). The aromatic ring and hydroxyl functional group of rosmarinic acid were shown to be important structural features for direct binding to Aβ amyloid, and a series of rosmarinic acid derivatives were developed on this basis [78,79,80].

## 4. Natural Products Reduce Tau Aggregation by Affecting Aggregate Formation, Disaggregation, and Key Enzyme Activity

Tau is a phosphoprotein with a natively unfolded conformation that functions to stabilize microtubules in axons. Microtubules form the cytoskeleton of the cell and are essential for maintaining the structural integrity of the cell and transporting nutrients from the soma down the axon to the synaptic terminal [129,130,131,132]. In the adult human central nervous system, tau proteins exist as six heterodimers containing 0, 1, or 2 amino-terminal inserts and 3 or 4 microtubule-binding repeat (3R or 4R) domains. Those repeats, containing 31 or 32 amino acid residues, form domains that stabilize microtubules and promote microtubule assembly [133,134]. The ability of tau to bind to microtubules is also regulated by the post-translational modification of proteins, including phosphorylation, glycosylation, glycation, ubiquitination, sumoylation, and nitration [131,135]. Tau has multiple kinase phosphorylation sites and its functions are partly regulated by its phosphorylation status [136,137]. In all neurodegenerative diseases associated with the tau protein, this protein is present in a hyperphosphorylated form, which is responsible for its aggregation and leads to neuronal dysfunction and death. The aggregation of tau is a multi-step process; the initial step is the formation of the β-sheets of tau, i.e., MTBR regions of tau stacked on top of each other [138]. Then, it forms dimers and trimers, followed by small soluble oligomers. These small soluble oligomers form twisted tau filaments called PHFs, which subsequently form neurofibrillary tangles (NFTs) [139]. Tau-associated diseases are known as tauopathies and include Alzheimer’s disease (AD), progressive supranuclear palsy (PSP), Pick’s disease, frontotemporal dementia (FTD), corticobasal degeneration, and variants of Parkinson’s disease (PD) and Lewy body dementia (LBD), for which NFTs are a common histopathological marker [135,140,141]. In addition, tau oligomers exhibit toxic effects in tauopathies prior to the formation of NFTs and are capable of potentiating neuronal damage, leading to neurodegeneration and traumatic brain injury [142]. Current drug strategies targeting the tau protein can be summarized as an inhibition of tau aggregation, inhibition of tau phosphorylation, reduction in tau levels and tau immunization [143]. Here, we review some of the compounds that have been shown to affect tau proteins.

**Figure 2:**
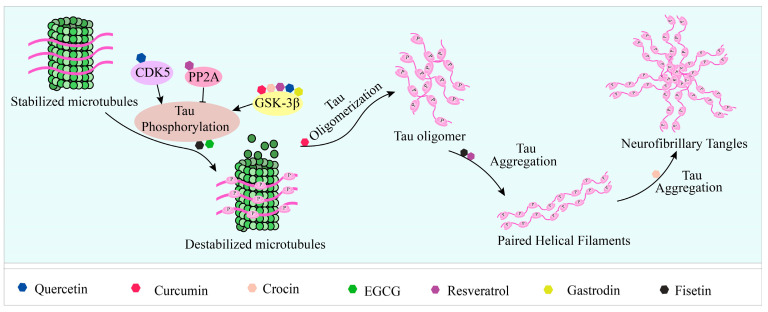
Natural products reduce tau aggregation, inhibit hyperphosphorylation, or act on formation processes. During the multistep process of tau aggregation, fisetin and EGCG reduce the phosphorylation level of tau. Curcumin, crocin, gastrodin, quercetin, and resveratrol can inhibit GSK3β activity and thus tau hyperphosphorylation. In addition, resveratrol and quercetin can inhibit tau phosphorylation by activating PP2A and inhibiting CDK5 activity, respectively. During the formation of tau oligomers, curcumin can inhibit its oligomerization. Fisetin and resveratrol can inhibit the accumulation of oligomers into PHFs, and curcumin can inhibit the further formation of NFTs from PHFs.

In mouse cortical neuronal cells expressing induced wild-type tau and in primary cortical neurons, Fistein significantly reduced phosphorylated tau levels, which was highly dependent on TFEB and Nrf2 activation and occurred via selective autophagy by its cargo receptors [144]. Tau K18 is a widely used model for full-length tau proteins, as they exhibit very similar physiological and pathological functions [145]. In vitro, Fisetin has been shown to limit the extent of tau K18-protofibril formation by inhibiting tau K18 aggregation (Figure 2), resulting in shorter and thinner tau protofibrils. In HEK293/tau441, treatment with Fisetin reduced tau oligomers and significantly decreased the ratio of insoluble-to-soluble tau protein [82]. And treatment with Fisetin (20 mg/kg, i.p., 2 weeks) significantly reduced p-tau levels at Ser413 induced by Aβ(1–42) injection (i.c.v.) in the hippocampus of mice [146].

Crocin has been shown to inhibit neuronal death [147,148], protect rats from brain ischemia/reperfusion injury, and enhance long-term potentiation, learning, recognition and memory [83,149,150]. In vitro, crocin inhibited the conversion of tau protein into more aggregated conformations during the fibrillation process by binding to its intermediate structures and inhibited 50% of tau aggregates at a dose of 100 µg/mL [151]. In rats, co-treatment with 25 mg/kg crocin significantly reversed the level of acrolein-induced phosphorylation of tau in the cerebral cortex by attenuating the active forms of ERK and JNK kinases [152]. GSK-3β is the most important protein kinase that regulates tau phosphorylation, when it is overactivated, tau is hyperphosphorylated. In PC12-htau cells, tau is hyperphosphorylated at Thr231 and Ser199/Ser202 compared to PC12 cells. In PC12-htau it has been shown that trans-Crocin 4 decreases the amount and phosphorylation of tau at the pThr231 and pSer199/Ser202 epitopes, and inhibits the active forms of GSK3β and ERK1/2 kinases [153].

Resveratrol (RES) has pharmacological properties with antioxidant, anti-inflammatory, hepatoprotective, anti-diabetic, and anti-tumor effects [154,155]. It is believed to have therapeutic potential in the treatment of neurodegenerative diseases. For example, treatment with RES reduced tau phosphorylation in the hippocampus of diabetic mice fed a high-fat diet, resulting in improved memory impairment [156]. An in vitro ThT fluorescence assay showed that RES inhibited tau aggregation, resulting in the formation of smaller aggregates rather than long fibers. Moreover, it prevented extracellular tau oligomers from binding to N2a cells, reduced tau propagation, and decreased the levels of phosphorylated tau and tau oligomers in the brains of PS19 mice [58]. In addition to GSK-3β, calmodulin-dependent protein kinase II (CaMKII) and phosphoserine/phosphothreonine protein phosphatase-2A (PP2A) are also important enzymes involved in the regulation of tau protein hyperphosphorylation [157,158,159,160,161]. RES inhibited formaldehyde-induced increasing phosphorylation of GSK-3β and CaMKII protein levels to prevent tau protein hyperphosphorylation, thereby protecting N2a cells from formaldehyde-induced damage [162]. PP2A dephosphorylates tau, preventing its microtubule dissociation and PHF formation. MID1 is a negative regulator of PP2A and mediates the ubiquitin-specific degradation of PP2A. The loss of its function results in increased PP2A protein levels and activity [163]. Both in vitro and in vivo, RES treatment destabilized the ubiquitin ligase MID1 and its mRNA, which directly interfered with the MID1–α4–PP2A degradation complex by decreasing MID1 protein expression, leading to an increase in microtubule-associated PP2A activity and the time- and dose- dependent dephosphorylation of tau [164]. Similarly, in the brain of CdCl2-treated rats, trans-resveratrol inhibited tau phosphorylation by activating PP2A and inhibiting GSK3β activity. In particular, the inhibition of GSK3β activity was mediated by AMPK-induced activation of the PI3K/Akt signaling pathway [165]. In addition, RES inhibited alum-induced tau hyperphosphorylation at the Ser396 site in rat hippocampal slices by decreasing ERK1/2 activation and increasing GSK-3β Ser9 phosphorylation [166]. In optic nerve head astrocytes (ONHAs) undergoing oxidative stress, pretreatment with resveratrol not only increased cell viability, but also reduced the levels of activated caspases and dephosphorylation of the tau protein at Ser422, thereby reducing caspase-mediated tau cleavage and neurogenic fiber tangle (NFT) formation [167].

As mentioned above, quercetin, curcumin, and EGCG exerted potent neuroprotective effects in inhibiting Aβ formation and attenuating Aβ toxicity. In addition to this, they also show protective effects in terms of lowering tau phosphorylation levels and reducing the levels of aggregated tau. Quercetin-3-O-glucuronide (Q3G), a major quercetin metabolite in human plasma, has been reported to have potential neuroprotective effects [168]. Pretreatment with 10 µM quercetin or Q3G inhibited okadaic acid (OA)-induced phosphorylation of the tau protein in SH-SY5Y. An oral administration of quercetin also effectively attenuated overexpression of the tau protein phosphorylation in the hippocampus of mice during HFD feeding. Further experiments demonstrated that this was due to the activation of AMPK and inhibition of GSK3β activation by enhancing phosphorylation at the Ser 9 residue [169]. Cell cycle protein-dependent kinase 5 (CDK5) is one of the kinases that affect tau phosphorylation, and overactivated CDK5 activity leads to an abnormal phosphorylation of tau [170]. Quercetin inhibited CDK5 activity, blocked the Ca2+–calpain–p25–CDK5 signaling pathway, and inhibited tau phosphorylation at four sites (Ser396, Ser199, Thr205, and Thr231), thus exhibiting significant neuroprotective effects on OA-induced Ht22 cells [171]. In vitro, quercetin was shown through ThT fluorometry to inhibit tau fibrillization and disassemble pre-formed aggregates of the tau protein [65]. Curcumin has long been shown to inhibit GSK-3β activity and prevent tau hyperphosphorylation, thereby protecting SH-SY5Y from Aβ-induced mitochondrial dysfunction [172,173]. In vitro, curcumin has been shown to inhibit the formation of tau β-sheets, inhibit tau fibrillation, and degrade formed tau filaments, thereby reducing the level of aggregated tau, with 20 µM curcumin leading to 75 ± 10% disaggregation of tau aggregates [51]. As for EGCG, in vitro, it blocked K18ΔK280 aggregation and inhibited the formation of potentially proteotoxic oligomeric tau species [174]. In primary neurons, phospho-tau (p-S396/404, p-S262, and p-T231) and total tau levels decreased after 24 h of 50 µM EGCG treatment, but mRNA levels of tau were not affected. This suggests that the reduction in tau was due to clearance rather than transcriptional repression [175]. Other studies have also shown that EGCG binds tau in its phosphorylation region with an affinity of the same order of magnitude as kinases (0.5 mM), preventing it from contacting the protein and thus playing a key role in preventing tau aggregation [73].

In addition to the compounds listed above, there are also many potential therapeutic agents in tauopathies. Gastrodin reduced tau phosphorylation levels of Ser396, Ser199, and Thr231, and inhibited GSK3β kinase activity levels in the brains of APP/PS1 transgenic mice [76]. Morin, a natural bioflavonoid, reduces tau hyperphosphorylation by inhibiting GSK3β activity and the CDK5 signaling pathway in mice [176,177]. The monoterpene 1,8-cineole (CIN), present in many plant essential oils, attenuated the abnormal phosphorylation levels of the tau protein at the thr205, thr181, and ser396 sites induced by AGEs in vitro and in vivo [178]. Macelignan, a sort of lignan derived from Myristica fragrans mace, reduced tau phosphorylation in tau-overexpressing cells and primary neurons of 3× AD-transgene mice. It also promoted PP2A activity in tau-overexpressing cells [126]. In addition, plant-derived nobiletin, beta boswellic acid, huperzine A, and caffeine exhibited the inhibition of tau hyperphosphorylation in different mouse models, respectively [179,180,181,182]. Isobavachalcone is the main component extracted from Psoralea corylifolia. In vitro, isobavachalcone can inhibit heparin-induced tau K18 aggregation and break down mature fibrils into shorter and smaller fibrils or short fragments. Furthermore, in N2a cells, it reduced the proportion of apoptosis caused by phosphatidylserine-induced tau K18 oligomer, from 40% to 10%. It also reduced the level of tau phosphorylation by regulating the levels of GSK3β and PP2A [183]. Limonoids (nimbin and salannin), isolated from neem fruit, were able to inhibit hTau40w aggregation and instead form thin, short, fragile tau fragments [184].

## 5. Natural Products Inhibit, Degrade, and Remodel α-Syn Fibrils to Reduce Accumulation and Toxicity

Alpha-synuclein (α-Syn) is an intrinsically disordered protein [185] that is abundant in the central nervous system [186] and transforms into cross-β-sheets rich amyloid by self-assembly under physiological conditions via partially folded intermediates and soluble oligomers [187]. Some aggregated species of α-Syn formed along the fibrillation are highly toxic and capable of interfering with the functions of different organelles such as mitochondria, endoplasmic reticulum, and plasma membrane [188,189,190]. Furthermore, it may increase oxidative stress, causing severe damages in dopaminergic cells [191,192]. Therefore, molecules that inhibit α-synuclein fibrillization and stabilize it in a non-toxic state can serve as therapeutic molecules that both prevent the accumulation of aggregated α-syn and maintain normal physiological concentrations of α-syn [193].

**Figure 3:**
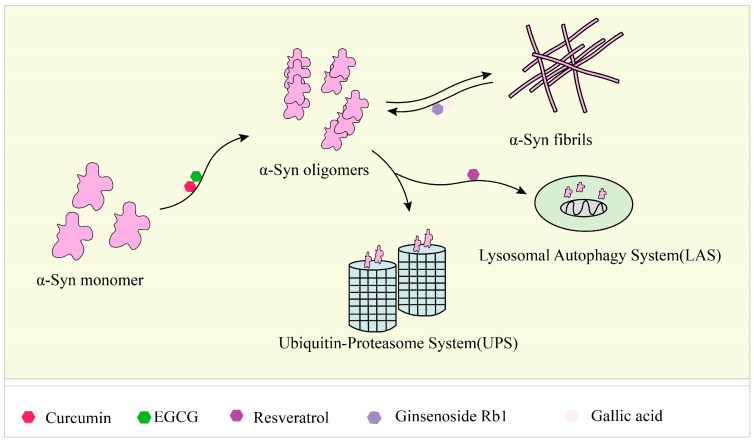
Responses of natural products to α-syn fibrils: inhibition, degradation, and remodeling. In the fibrosis of α-syn, curcumin and EGCG can inhibit its conversion from a monomer to an oligomer, and ginsenoside Rb1 and gallic acid can degrade the formed fibrils. Resveratrol can induce the autophagic degradation of α-syn.

Studies have identified small molecules, nanoparticles, peptides, and polymers that have the ability to inhibit α-synuclein fibril formation or destabilize preformed α-syn fibrils (Figure 3). Curcumin has been mentioned above for its significant inhibitory effect on the formation of aggregates of Aβ and tau [194,195,196,197]. Curcumin has also been shown to inhibit the aggregation of α-syn in vitro and attenuate the toxicity of α-syn oligomers in cells [52,53]. In addition, curcumin prevented lipopolysaccharide-induced increases in α-syn gene expression in rats [198]. Due to the instability of curcumin in solution, stable curcumin analogues have raised some concerns. Curcumin pyrazole and its derivative (N-(3-nitrophenylpyrazole) curcumin inhibited the aggregation, protofibrosis, and toxicity of α-syn. Through biochemical, biophysical, and cell-based assays, both have been found to exhibit significant efficacy not only in arresting fibrillization and destroying pre-formed fibrils, but also in preventing formation of the A11 conformation in proteins, which can have toxic effects [199]. EGCG is another natural product that has received particular attention for targeting α-syn fibrillization due to its high availability and low toxicity [200,201]. In vitro, EGCG effectively inhibited α-syn fibrillogenesis by binding to naturally unstructured α-syn monomers and preventing their conversion into stable, β-sheet-rich structures. Instead, it promoted the formation of a novel non-structural, non-toxic α-synuclein [66]. In the rat immortalized oligodendrocyte cell line, OLN-93, EGCG immobilized the C-terminal region, moderately reduced the degree of oligomer binding to the membrane, and inhibited the ability of pre-formed oligomers to permeabilize vesicles and induce cytotoxicity [202]. ‘Active’ oligomers (AOs), characterized as a meta-stable and β-sheet-free species, exhibit rapid self-assembly into the radiating amyloid fibrils (RAFs) on the liposome surface, leading to drastic disruption of the membrane structures [203]. EGCG suppressed the membrane-disrupting radiating amyloid fibril formation on the surface of liposomal membranes, thus protecting the cells that can be readily affected by Aos [204]. According to the results of a molecular dynamics simulation, EGCG can disrupt the β-sheet structure and reduce the β-sheet content to remodel α-syn fibrils [205,206].

There is evidence that alterations in the autophagy lysosomal pathway of α-synuclein degradation may be preferentially involved in neuronal death and contribute to the pathogenesis of PD [207,208]. RES-activated SIRT1, deacetylated microtubule-associated protein 1 light chain 3 (LC3), and caused the autophagic degradation of α-syn in dopaminergic neurons [84]. Studies have shown that ginsenoside Rb1 effectively inhibited α-syn fibrillation, with an inhibition rate of approximately 90% at 25 µM and incubation for two days. Additionally, Rb1 exhibited a strong ability to decompose preformed fibrils and inhibit the seeded polymerization of α-syn [85]. In vitro thioflavin T fluorescence assays and transmission electron microscopy imaging results showed that GA can inhibit the formation of amyloid fibrils by α-syn and disaggregate preformed α-syn amyloid fibrils. For soluble non-toxic oligomers without β-sheet content, GA can bind to them to stabilize their structure [84,86]. Triptolide (T10) is a monomeric compound isolated from Tripterygium wilfordii Hook f (TWHF). It has anti-inflammatory and anti-tumor activities, as well as neuroprotective effects [209,210]. In neuronal cells, T10 decreased the expression level of α-syn and acted as an autophagy inducer to promote the degradation of α-syn without disturbing lysosomal function [211].

In addition to the above, other compounds have been found to have effects on α-syn aggregation in vitro. For example, the components of saffron, crocin-1, crocin-2, and crocetin, inhibited α-syn aggregation, and dissociated α-syn fibrils [149]. The compounds in *Rose damascena* can inhibit α-syn fibrillation and oligomer toxicity [212]. In addition, the combined action of the compounds offers a new possibility. Protocatechuic acid (PCA) and hydroxytyrosol (HT) were able to reduce α-syn toxicity. When PCA (100 μM) and HT (100 μM) were used in combination, they showed a higher inhibition of α-syn protofibril formation and destabilization of α-syn fibrils, of 88% and 62%, respectively [213].

## 6. EGCG and Ellagic Acid Dose-Dependently Inhibit Htt Protein Aggregates and Increase Cell Viability

**Figure 4:**
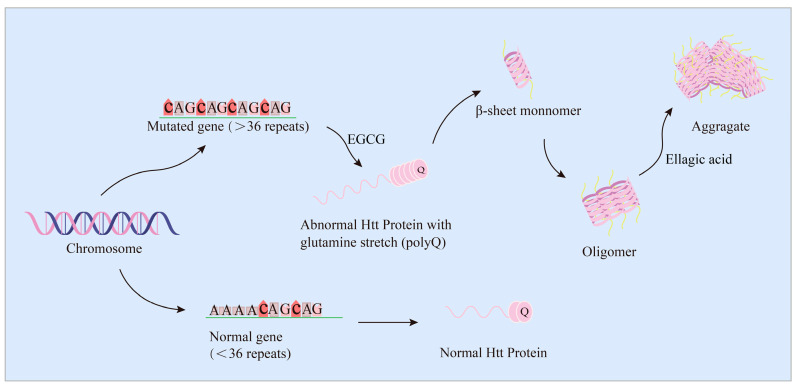
EGCG and ellagic acid act on the Htt lesion process. EGCG specifically binds polyglutamine poly Q sequence and inhibits the formation of small oligomers. EA inhibits the formation of HD53Q aggregates and restores cell viability.

Huntingtin protein (Htt protein) is a key functional protein in the pathogenesis of Huntington’s disease (HD) [214,215]. Under normal physiological conditions, the Htt protein can interact with many proteins to perform biological functions in cells such as protein transport, vesicular trafficking, postsynaptic signaling, transcriptional regulation, and the inhibition of apoptosis [216]. In pathological conditions, the HTT gene, exon 1 CAG repeats are increased and the polyglutamine (polyQ) is extended and expanded, resulting in HTT mutation (mHTT) [217]. The degree of Htt fibrosis is directly related to the length of polyQ, which exceeds 35 polyQ as a critical value [218,219]. The expanded and extended polyQ forms oligomers, protofibrils, and fibrillated amyloid due to the folding of β-sheet structures, thus causing increased free radicals, mitochondrial dysfunction, and inflammatory factor production, leading to disease onset [216,220]. As shown in Figure 4, there are some traditional therapeutic approaches based on natural products that have been shown to have broad therapeutic benefits for mHTT-induced aggregates in both in vitro and in vivo models [220,221,222].

In an in vitro protein purification assay, EGCG was able to inhibit 51 glutamine-producing aggregates in a dose-dependent manner at a semi-inhibitory concentration of 1 μM after 16 h of incubation [223]. EGCG inhibited the formation of 53 glutamine-induced small oligomers in vitro by stimulating the formation of larger-diameter (120–200 nm) macromolecules. When the concentration of EGCG was five times the molarity of the aggregation reaction, EGCG could bind to non-structural polyQ sequences and interfere with the formation of polyQ aggregates [223]. EGCG not only reduced damage to aggregates and reduced the number of aggregates in the yeast (GFP-HDQ72) model, but it also reduced photoreceptor degeneration and motor damage in the HDQ93 *Drosophila* model [223,224]. In the study of lipid membrane interactions, it was shown that the ability of EGCG to regulate aggregates was enhanced by the presence of lipid vesicles [225]. Ellagic acid (2,3,7,8-tetrahydroxybenzopyrano (5,4,3-cde) benzophyran-5–10-dione, EA) is a polyphenolic antioxidant found in pomegranates, raspberries, strawberries, cranberries, and walnuts. In vitro, ellagic acid (160 µM) was observed via transmission electron microscopy to not only inhibit the formation of HD53Q amyloid, but also increase the viability of 3 μM HD53Q-induced neurotoxic cells by 40.3% when 9 μM ellagic acid was added [81]. In vivo, high doses of ellagic acid significantly reduced mHTT aggregates in the striatum and cortex of R6/2 mice shown through EM48-immunostaining (about 50–70% reduction) [81]. Harmine is an alkaloid plant antioxidant that solubilized 103Q-htt aggregates in yeast in vivo in a dose-dependent manner (25 μg/mL) and restored cell viability by reducing the damage caused by oxidative stress [226].

## 7. EGCG Directly Targets the Structural Domain of the FUS Protein to Inhibit Aggregate Formation

**Figure 5:**
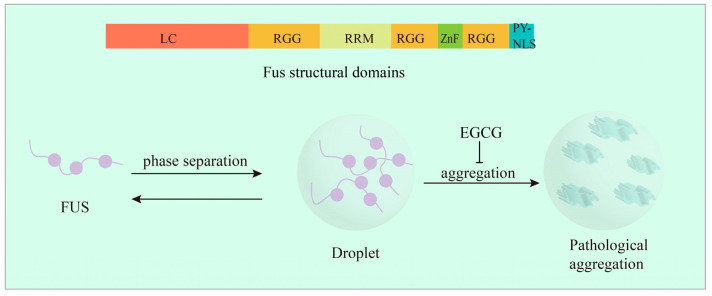
FUS structural domain and the liquid–solid phase transition process. EGCG binds directly to the RG/RGG structural domain of FUS to promote FUS droplet formation and inhibit the abnormal phase transition of FUS proteins from membraneless organelles to toxic aggregates and protofibrils.

FUS is a sarcoma fusion protein that has been identified as a cause or risk factor for the neurodegenerative diseases amyotrophic lateral sclerosis (ALS), idiopathic tremor, and the rare frontotemporal lobar degeneration (FTLD) [227]. FUS is a DNA/RNA-binding protein that is mainly localized in the nucleus and has a total of 526 amino acids. Under normal physiological function, FUS is capable of undergo liquid–liquid phase separation, a process in which a supersaturated solution spontaneously forms two physical phases of different densities that can stably coexist [228]. FUS has been shown to be involved in the DNA repair process as a function of instantaneous liquid–liquid phase separation [229]. However, FUS is highly susceptible to self-aggregation, and in vitro or in pathological models, FUS results in different states such as amyloid-hydrogel-like or aggregated solid forms due to self-aggregation or abnormal liquid phase mass changes [230]. As shown in Figure 5, the structural domain of FUS is roughly divided into seven parts: an N-terminal serine-rich LC disordered domain (NTD), followed by tyrosine, glycine, and glutamine (QGSY) residues, followed by three arginine–glycine–glycine repeats (RGG) and an RNA recognition motif (RRM), and finally a zinc finger domain (ZnF) and a proline–tyrosine nuclear localization sequence (PY-NLS) [231]. Different structures of FUS play different roles, and the occurrence of a liquid–liquid phase separation in FUS is regulated by several structural domains, especially the LC region, which dominates the liquid–liquid phase separation and anomalous phase transition of FUS. Recent studies have shown that EGCG directly binds to the RG/RGG structural domain of FUS to promote FUS droplet formation, and arginine methylation enhances this effect [232]. In an in vitro model of purified FUS (RGG-3PY), EGCG exhibited high affinity to RGG-3PY at both high micromolar and millimolar levels. The methylation of arginine was detected via NMR spectroscopy to enhance the binding of EGCG to the FUS protein, thereby inhibiting the abnormal phase transition of FUS protein from membraneless organelles to toxic aggregates and protofibrils [232]. There are few natural products that can directly affect the structure and formation process of FUS protein aggregates and restore their normal phase transition function, and this will be a key issue in future research. The study of natural products that inhibit the self-aggregation of FUS proteins will also provide a new idea and theoretical basis for the treatment of major diseases such as ALS and FTLD.

## 8. Conclusions and Perspectives

Neurodegenerative diseases, which lead to progressive neuronal cell damage and the loss of neuronal connections, ultimately resulting in impaired mobility, memory loss, and cognitive impairment, have become a common challenge for humanity. Protein aggregation due to misfolding and oligomerization is one of the common hallmarks of many neurodegenerative diseases, and many scientists have conducted extensive research to explore the morphology, structure, and molecular mechanisms leading to aggregation in an attempt to find ways to inhibit protein formation and reduce protein aggregation.

The advantages of natural products are as follows. Natural products are a starting point for drug discovery. Natural products are usually found in plants or fruits, and the raw materials are easily available. In addition, the molecular scaffold of natural products is rich and diverse, which can be used to rationally design drugs using electronic computer-aided designs.

The disadvantages of natural products are that natural products have low monomer availability, a complex and lengthy extraction process, and high depletion. Secondly, natural products have low bioavailability, limited water solubility, unstable physicochemical properties, rapid metabolism, and they cross the blood–brain barrier (BBB) [233,234,235,236,237,238]. However, natural products are grown in nature, and because their molecular structures are easy to study and modify and they have low toxicity and few side effects, they are now receiving a great deal of attention as good candidates for safe treatment at the preclinical stage of disease. The unique molecular structural features of natural compounds play a key role in inhibiting amyloid formation, such as curcumin, of which the hydrophobic interaction and hydrogen bonding in the symmetrical molecular structure can deform the β-sheet structure, and the π-stacking between the aromatic residues that bind Aβ leads to a reduction in the β-sheet structure, thus inhibiting the formation of Aβ aggregates. CA and FA have similar effects. EGCG binds to non-structural polyQ sequences, reducing the number of polyQ, interfering with the formation of polyQ aggregates, and reducing the probability of developing HD. EGCG directly binds to the RG/RGG structural domain of FUS, promoting the formation of FUS droplets and ensuring the normal physiological function of FUS. To inhibit the formation of protein aggregates, many studies have also been conducted on compounds that can affect gene expression levels and thus key enzyme activity levels, such as EGCG and resveratrol, which can affect the phosphorylation of the tau protein by down-regulating GSK3β expression levels, reducing oligomer formation and protofibrillation. Curcumin, which is rich in biological activity, inhibits the deposition of Aβ aggregates by binding to the N-terminal end of Aβ and reduces the level of BACE1 enzyme activity by modulating the ERβ and NFκB pathways, thereby attenuating the neurological damage of Aβ aggregation. In addition to directly targeting the misfolding and the aggregation process of various amyloid proteins, some natural products have been shown to act downstream of protein aggregation to prevent the toxic consequences of misfolded protein accumulation. Examples include targeting the secondary processes induced by the accumulation of misfolded proteins, inflammation, oxidative stress, and dysregulation of proteostasis. Although much work has been carried out to investigate the effects of natural products on pathogenic protein aggregation, there are still shortcomings. For example, some natural products are metabolically unstable and have low bioavailability, which needs to be strengthened in the development of analogues and derivatives. In addition, experimental studies on the effects of natural products on protein aggregation are not detailed enough, and it is still worthwhile to develop and explore the mechanism and analyze how to reduce oligomeric and fibrillated proteins. More importantly, there is a wide variety of natural products and a huge number of them, and in future research, selective and focused systematic screening of natural product libraries is of great importance. Neurodegenerative diseases cause immense physical and psychological suffering, and the use of natural products as a preventive intervention should be further explored in clinical research. More research is therefore needed on how to best utilize natural products to treat and prevent some of the current debilitating chronic diseases.

## Figures and Tables

**Table 1:** Natural products target pathogenic protein aggregates in neurodegenerative diseases.

Compound	Chemical Structure Formula	Source	Target *	Effect on Aggregates	Reference
Curcumin	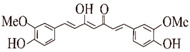	Ginger	Aβ ^②④⑤⑥^ tau ^①②⑥^ α-Syn ^①⑥^	(I) Inhibits formation (II) Disassembles aggregates	[46,47,48,49,50,51,52,53]
Resveratrol (RES)	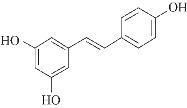	Berries, grapes, and peanuts	Aβ ^②⑥^ tau ^①②⑥^ α-Syn ^⑥^	(I) Inhibits formation (II) Disassembles aggregates	[54,55,56,57,58,59]
Ferulic acid (FA)	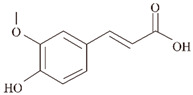	Plants	Aβ ^④⑥^	Inhibits formation	[60,61]
Caffeic acid (CA)	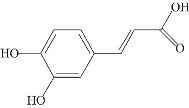	Plants, fruits, wine, coffee, olive oil, and legumes	Aβ ^②⑥^	Inhibits formation	[62]
Quercetin	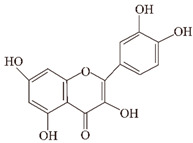	Apples, berries, grapes, cherries, broccoli, and etc.	Aβ ^②^, tau ^①②⑥^	(Ⅰ) Inhibits formation (Ⅱ) Disassembles aggregates	[63,64,65]
Epigallocatechin-3-gallocatechin (EGCG)	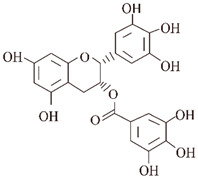	Green tea	Aβ ^①^, tau ^①^, α-Syn ^①^,Htt ^①③^, and FUS ^①^	(Ⅰ) Disassembles aggregates (Ⅱ) Inhibits formation (Ⅲ) Inhibits toxicity (Ⅳ) Remodels aggregates	[66,67,68,69,70,71,72,73]
Salvianolic acid A	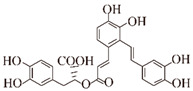	Chinese herb *Salvia miltiorrhiza*	Aβ ^②^	(Ⅰ) Inhibits formation (Ⅱ) Disassembles aggregates	[74]
Salvianolic acid B	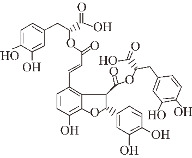	Chinese herb *Salvia miltiorrhiza*	Aβ ^②^	(Ⅰ) Inhibits formation (Ⅱ) Disassembles aggregates	[75]
Gastrodin	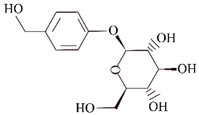	Chinese traditional medicinal herbs tianma	Aβ ^②⑥^	(Ⅰ) Inhibits formation (Ⅱ) Promotes clearance	[76]
Isoorientin	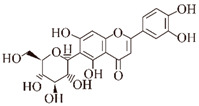	Plants	Aβ ^②⑥^	Inhibits formation	[77]
Rosmarinic acid	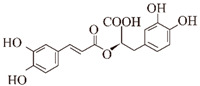	Plants	Aβ ^⑥^	Disassembles aggregates	[78,79,80]
Ellagic acid	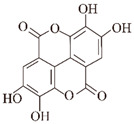	Pomegranates, raspberries, strawberries, etc.	Htt ^②⑥^	Inhibits formation	[81]
Fisetin	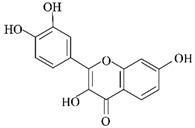	Fruits and vegetables	tau ^①②⑥^	(Ⅰ) Inhibits formation (Ⅱ) Reduces insoluble protein	[82]
Crocin	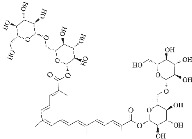	*Crocus sativus* L.	tau ^①②⑥^	Inhibits formation	[83]
Ginsenoside Rb1	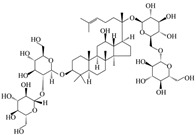	Ginseng	α-Syn ^①^	(Ⅰ) Inhibits formation (Ⅱ) Disassembles aggregates	[84]
Gallic acid	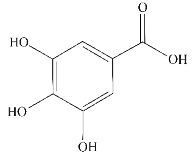	Tea	α-Syn ^①^	(Ⅰ) Inhibits formation (Ⅱ) Disassembles aggregates	[85,86]

* Experimental models used for the target proteins: ^①^ in-vitro-purified proteins; ^②^ in mammalian cells; ^③^ in yeast cells; ^④^ in *Caenorhabditis elegans*; ^⑤^ in *Drosophlia*; ^⑥^ in mice.

## Data Availability

The data presented in this study are available in this paper.

## References

[1] Sharma N., Tan M.A., An S.S.A. (2021). Phytosterols: Potential metabolic modulators in neurodegenerative diseases. Int. J. Mol. Sci..

[2] Gauthier S., Rosa-Neto P., Morais J.A., Webster C. (2021). World Alzheimer Report 2021 Journey through the Diagnosis of Dementia.

[3] Dorsey E.R., Sherer T., Okun M.S., Bloem B.R. (2018). The Emerging Evidence of the Parkinson Pandemic. J. Parkinsons. Dis..

[4] Cerri S., Mus L., Blandini F. (2019). Parkinson’s Disease in Women and Men: What’s the Difference?. J. Parkinsons. Dis..

[5] Pringsheim T., Wiltshire K., Day L., Dykeman J., Steeves T., Jette N. (2012). The incidence and prevalence of Huntington’s disease: A systematic review and meta-analysis. Mov. Disord..

[6] Caron N.S., Dorsey E.R., Hayden M.R. (2018). Therapeutic approaches to Huntington disease: From the bench to the clinic. Nat. Rev. Drug Discov..

[7] Mathis S., Goizet C., Soulages A., Vallat J.M., Masson G.L. (2019). Genetics of amyotrophic lateral sclerosis: A review. J. Neurol. Sci..

[8] Hassan S.S.U., Samanta S., Dash R., Karpinski T.M., Habibi E., Sadiq A., Ahmadi A., Bunagu S. (2022). The neuroprotective effects of fisetin, a natural flavonoid in neurodegenerative diseases: Focus on the role of oxidative stress. Front. Pharmacol..

[9] Glenner G.G., Wong C.W. (1984). Alzheimer’s disease: Initial report of the purification and characterization of a novel cerebrovascular amyloid protein. Biochem. Biophys. Res. Commun..

[10] Grundke-Iqbal I., Iqbal K., Quinlan M., Tung Y.C., Zaidi M.S., Wisniewski H.M. (1986). Microtubule-associated protein tau. A component of Alzheimer paired helical filaments. J. Biol. Chem..

[11] Spillantini M.G., Schmidt M.L., Lee V.M., Trojanowski J.Q., Jakes R., Goedert M. (1997). Alpha-synuclein in Lewy bodies. Nature.

[12] Polymeropoulos M.H., Lavedan C., Leroy E., Ide S.E., Dehejia A., Dutra A., Pike B., Root H., Rubenstein J., Boyer R. (1997). Mutation in the alpha-synuclein gene identified in families with Parkinson’s disease. Sci. N. Y..

[13] Bates G. (2003). Huntingtin aggregation and toxicity in Huntington’s disease. Lancet.

[14] Taylor J.P., Brown R.H., Cleveland D.W. (2016). Decoding ALS: From genes to mechanism. Nature.

[15] Salem M.A., Perez de Souza L., Serag A., Fernie A.R., Farag M.A., Ezzat S.M., Alseekh S. (2020). Metabolomics in the context of plant natural products research: From sample preparation to metabolite analysis. Metabolites.

[16] Mohd Sairazi N.S., Sirajudeen K.N.S. (2020). Natural products and their bioactive compounds: Neuroprotective potentials against neurodegenerative diseases. Evid. Based Complement. Alternat. Med..

[17] Zhang L., Song J., Kong L., Yuan T., Li W., Zhang W., Hou B., Lu Y., Du G. (2020). The strategies and techniques of drug discovery from natural products. Pharmacol. Therapeut..

[18] Essa M.M., Vijayan R.K., Castellano-Gonzalez G., Memon M.A., Braidy N., Guillemin G.J. (2012). Neuroprotective effect of natural products against Alzheimer’s disease. Neurochem. Res..

[19] Chen X., Drew J., Berney W., Lei W. (2021). Neuroprotective natural products for Alzheimer’s disease. Cells.

[20] Wang Z., He C., Shi J.S. (2020). Natural products for the treatment of neurodegenerative diseases. Curr. Med. Chem..

[21] Taylor E., Kim Y., Zhang K., Chau L., Nguyen B.C., Rayalam S., Wang X. (2022). Antiaging Mechanism of natural compounds: Effects on autophagy and oxidative stress. Molecules.

[22] Scrivo A., Bourdenx M., Pampliega O., Cuervo A.M. (2018). Selective autophagy as a potential therapeutic target for neurodegenerative disorders. Lancet Neurol..

[23] Cui X., Lin Q., Liang Y. (2020). Plant-derived antioxidants protect the nervous system from aging by inhibiting oxidative stress. Front. Aging Neurosci..

[24] Lee J.H., Ahn N.H., Choi S.B., Kwon Y., Yang S.H. (2021). Natural products targeting amyloid beta in Alzheimer’s disease. Int. J. Mol. Sci..

[25] Witter S., Samoson A., Vilu R., Witter R. (2020). Screening of nutraceuticals and plant extracts for inhibition of amyloid-β fibrillation. J. Alzheimers Dis..

[26] Noori T., Dehpour A.R., Sureda A., Sobarzo-Sanchez E., Shirooie S. (2021). Role of natural products for the treatment of Alzheimer’s disease. Eur. J. Pharmacol..

[27] Rocha-Gonzalez H.I., Ambriz-Tututi M., Granados-Soto V. (2008). Resveratrol: A natural compound with pharmacological potential in neurodegenerative diseases. CNS Neurosci. Ther..

[28] Rao T., Tan Z., Peng J., Guo Y., Chen Y., Zhou H., Ouyang D. (2019). The pharmacogenetics of natural products: A pharmacokinetic and pharmacodynamic perspective. Pharmacol. Res..

[29] Rodrigues T., Reker D., Schneider P., Schneider G. (2016). Counting on natural products for drug design. Nat. Chem..

[30] Al-Rahbi B., Zakaria R., Othman Z., Hassan A., Ahmad A.H. (2014). Protective effects of tualang honey against oxidative stress and anxiety-like behaviour in stressed ovariectomized rats. Internat. Schol. Res. Not..

[31] Ryu S., Koo S., Ha K.-T., Kim S. (2016). Neuroprotective effect of Korea red ginseng extract on 1-methyl-4-phenyl-pyridinium-induced apoptosis in PC12 cells. Anim. Cells Syst..

[32] Feng L., Zhang L. (2019). Resveratrol suppresses abeta-induced microglial activation through the TXNIP/TRX/NLRP3 signaling pathway. DNA Cell Biol..

[33] Yang X., Qiang X., Li Y., Luo L., Xu R., Zheng Y., Cao Z., Tan Z., Deng Y. (2017). Pyridoxine-resveratrol hybrids Mannich base derivatives as novel dual inhibitors of AChE and MAO-B with antioxidant and metal-chelating properties for the treatment of Alzheimer’s disease. Bioorg. Chem..

[34] Carrizzo A., Forte M., Damato A., Trimarco V., Salzano F., Bartolo M., Maciag A., Puca A.A., Vecchione C. (2013). Antioxidant effects of resveratrol in cardiovascular, cerebral and metabolic diseases. Food Chem. Toxicol..

[35] Li S.Y., Wang X.B., Kong L.Y. (2014). Design, synthesis and biological evaluation of imine resveratrol derivatives as multi-targeted agents against Alzheimer’s disease. Eur. J. Med. Chem..

[36] Gomes B.A.Q., Silva J.P.B., Romeiro C.F.R., Dos Santos S.M., Rodrigues C.A., Goncalves P.R., Sakai J.T., Mendes P.F.S., Varela E.L.P., Monteiro M.C. (2018). Neuroprotective mechanisms of resveratrol in Alzheimer’s disease: Role of SIRT1. Oxid. Med. Cell. Longev..

[37] Zwolak I. (2021). Epigallocatechin gallate for management of heavy metal-induced oxidative stress: Mechanisms of action, efficacy, and concerns. Int. J. Mol. Sci..

[38] Saeed A.A., Genové G., Li T., Lütjohann D., Olin M., Mast N., Pikuleva I.A., Crick P., Wang Y., Griffiths W. (2014). Effects of a disrupted blood-brain barrier on cholesterol homeostasis in the brain. J. Biol. Chem..

[39] Andrade S., Nunes D., Dabur M., Ramalho M.J., Pereira M.C., Loureiro J.A. (2023). Therapeutic potential of natural compounds in neurodegenerative diseases: Insights from clinical trials. Pharmaceutics.

[40] Turner R.S., Thomas R.G., Craft S., van Dyck C.H., Mintzer J., Reynolds B.A., Brewer J.B., Rissman R.A., Raman R., Aisen P.S. (2015). A randomized, double-blind, placebo-controlled trial of resveratrol for Alzheimer disease. Neurology.

[41] Zia A., Farkhondeh T., Pourbagher-Shahri A.M., Samarghandian S. (2021). The role of curcumin in aging and senescence: Molecular mechanisms. Biomed. Pharmacother..

[42] Jabczyk M., Nowak J., Hudzik B., Zubelewicz-Szkodzińska B. (2021). Curcumin and its potential impact on microbiota. Nutrients.

[43] Tomeh M.A., Hadianamrei R., Zhao X. (2019). A review of curcumin and its derivatives as anticancer agents. Int. J. Mol. Sci..

[44] Han Y., Chen R., Lin Q., Liu Y., Ge W., Cao H., Li J. (2021). Curcumin improves memory deficits by inhibiting HMGB1-RAGE/TLR4-NF-kappaB signalling pathway in APPswe/PS1dE9 transgenic mice hippocampus. J. Cell. Mol. Med..

[45] Du S., Zhang Y., Yang J., Liu X., Wang Y., Xu B., Jia J. (2019). Curcumin alleviates beta amyloid-induced neurotoxicity in HT22 Cells via upregulating SOD2. J. Mol. Neurosci..

[46] Fu Z., Aucoin D., Ahmed M., Ziliox M., Van Nostrand W.E., Smith S.O. (2014). Capping of abeta42 oligomers by small molecule inhibitors. Biochemistry..

[47] Zhao L.N., Chiu S.W., Benoit J., Chew L.Y., Mu Y. (2012). The effect of curcumin on the stability of abeta dimers. J. Phys. Chem. B..

[48] Thapa A., Vernon B.C., De la Pena K., Soliz G., Moreno H.A., Lopez G.P., Chi E.Y. (2013). Membrane-mediated neuroprotection by curcumin from amyloid-beta-peptide-induced toxicity. Langmuir..

[49] Caesar I., Jonson M., Nilsson K.P., Thor S., Hammarstrom P. (2012). Curcumin promotes A-beta fibrillation and reduces neurotoxicity in transgenic *Drosophila*. PLoS ONE.

[50] Chainoglou E., Hadjipavlou-Litina D. (2020). Curcumin in health and diseases: Alzheimer’s disease and curcumin analogues, derivatives, and hybrids. Int. J. Mol. Sci..

[51] Rane J.S., Bhaumik P., Panda D. (2017). Curcumin Inhibits Tau Aggregation and Disintegrates Preformed Tau Filaments in vitro. J. Alzheimers. Dis..

[52] Ahmad B., Lapidus L.J. (2012). Curcumin prevents aggregation in α-synuclein by increasing reconfiguration rate. J. Biol. Chem..

[53] Liu Z., Yu Y., Li X., Ross C.A., Smith W.W. (2011). Curcumin protects against A53T alpha-synuclein-induced toxicity in a PC12 inducible cell model for Parkinsonism. Pharmacol. Res..

[54] Sanchez-Melgar A., Izquierdo-Ramirez P.J., Grinan-Ferre C., Pallas M., Martin M., Albasanz J.L. (2022). Neuroprotective effects of resveratrol by modifying cholesterol metabolism and abeta processing in SAMP8 Mice. Int. J. Mol. Sci..

[55] Rahman M.H., Akter R., Bhattacharya T., Abdel-Daim M.M., Alkahtani S., Arafah M.W., Al-Johani N.S., Alhoshani N.M., Alkeraishan N., Alhenaky A. (2020). Resveratrol and neuroprotection: Impact and its therapeutic potential in Alzheimer’s disease. Front. Pharmacol..

[56] Al-Edresi S., Alsalahat I., Freeman S., Aojula H., Penny J. (2020). Resveratrol-mediated cleavage of amyloid beta(1-42) peptide: Potential relevance to Alzheimer’s disease. Neurobiol. Aging..

[57] Chen Y., Shi G.W., Liang Z.M., Sheng S.Y., Shi Y.S., Peng L., Wang Y.P., Wang F., Zhang X.M. (2019). Resveratrol improves cognition and decreases amyloid plaque formation in Tg6799 mice. Mol. Med. Rep..

[58] Sun X.Y., Dong Q.X., Zhu J., Sun X., Zhang L.F., Qiu M., Yu X.L., Liu R.T. (2019). Resveratrol rescues tau-induced cognitive deficits and neuropathology in a mouse model of tauopathy. Curr. Alzheimer Res..

[59] Guo Y.J., Dong S.Y., Cui X.X., Feng Y., Liu T., Yin M., Kuo S.H., Tan E.K., Zhao W.J., Wu Y.C. (2016). Resveratrol alleviates MPTP-induced motor impairments and pathological changes by autophagic degradation of α-synuclein via SIRT1-deacetylated LC3. Mol. Nutr. Food. Res..

[60] Ono K., Hirohata M., Yamada M. (2005). Ferulic acid destabilizes preformed beta-amyloid fibrils in vitro. Biochem. Biophys. Res. Commun..

[61] Salamanova E., Atanasova M., Dimitrov I., Doytchinova I. (2021). Effects of curcumin and ferulic acid on the folding of amyloid-beta peptide. Molecules.

[62] Arai T., Ohno A., Mori K., Kuwata H., Mizuno M., Imai K., Hara S., Shibanuma M., Kurihara M., Miyata N. (2016). Inhibition of amyloid fibril formation and cytotoxicity by caffeic acid-conjugated amyloid-beta C-terminal peptides. Bioorg. Med. Chem. Lett..

[63] Alghamdi A., Birch D.J.S., Vyshemirsky V., Rolinski O.J. (2022). Impact of the Flavonoid Quercetin on beta-amyloid aggregation revealed by intrinsic fluorescence. J. Phys. Chem. B.

[64] Godoy J.A., Lindsay C.B., Quintanilla R.A., Carvajal F.J., Cerpa W., Inestrosa N.C. (2017). Quercetin exerts differential neuroprotective effects against H2O2 and abeta aggregates in hippocampal neurons: The role of mitochondria. Mol. Neurobiol..

[65] Kumar S., Krishnakumar V.G., Morya V., Gupta S., Datta B. (2019). Nanobiocatalyst facilitated aglycosidic quercetin as a potent inhibitor of tau protein aggregation. Int. J. Biol. Macromol..

[66] Ehrnhoefer D.E., Bieschke J., Boeddrich A., Herbst M., Masino L., Lurz R., Engemann S., Pastore A., Wanker E.E. (2008). EGCG redirects amyloidogenic polypeptides into unstructured, off-pathway oligomers. Nat. Struct. Mol. Biol..

[67] Palhano F.L., Lee J., Grimster N.P., Kelly J.W. (2013). Toward the molecular mechanism(s) by which EGCG treatment remodels mature amyloid fibrils. J. Am. Chem. Soc..

[68] Ahmed R., VanSchouwen B., Jafari N., Ni X., Ortega J., Melacini G. (2017). Molecular Mechanism for the (-)-Epigallocatechin Gallate-Induced Toxic to Nontoxic Remodeling of Aβ Oligomers. J. Am. Chem. Soc..

[69] Bieschke J., Russ J., Friedrich R.P., Ehrnhoefer D.E., Wobst H., Neugebauer K., Wanker E.E. (2010). EGCG remodels mature alpha-synuclein and amyloid-beta fibrils and reduces cellular toxicity. PNAS..

[70] Teng Y., Zhao J., Ding L., Ding Y., Zhou P. (2019). Complex of EGCG with Cu(II) suppresses amyloid aggregation and Cu(II)-Induced cytotoxicity of alpha-synuclein. Molecules.

[71] Choi J.S., Braymer J.J., Nanga R.P., Ramamoorthy A., Lim M.H. (2010). Design of small molecules that target metal-abeta species and regulate metal-induced abeta aggregation and neurotoxicity. Proc. Natl. Acad. Sci. USA.

[72] DeToma A.S., Krishnamoorthy J., Nam Y., Lee H.J., Brender J.R., Kochi A., Lee D., Onnis V., Congiu C., Manfredini S. (2014). Synthetic flavonoids, aminoisoflavones: Interaction and reactivity with metal-free and metal-associated amyloid-beta species. Chem. Sci..

[73] Gueroux M., Fleau C., Slozeck M., Laguerre M., Pianet I. (2017). Epigallocatechin 3-gallate as an inhibitor of tau phosphorylation and aggregation: A molecular and structural insight. J. Prev. Alzheimers Dis..

[74] Tang Y., Huang D., Zhang M.H., Zhang W.S., Tang Y.X., Shi Z.X., Deng L., Zhou D.H., Lu X.Y. (2016). Salvianolic Acid B inhibits abeta generation by modulating BACE1 activity in SH-SY5Y-APPsw cells. Nutrients.

[75] Jianwei Liu Y.W. (2020). Jing Guo, Jinyan Sun, Qinfeng Sun, Salvianolic Acid B improves cognitive impairment by inhibiting neuroinflammation and decreasing Aβ level in porphyromonas gingivalis-infected mice. Aging.

[76] Zeng Y.-Q., Gu J.-H., Chen L., Zhang T.-T., Zhou X.-F. (2021). Gastrodin as a multi-target protective compound reverses learning memory deficits and AD-like pathology in APP/PS1 transgenic mice. J. Funct. Foods.

[77] Tan X., Liang Z., Li Y., Zhi Y., Yi L., Bai S., Forest K.H., Nichols R.A., Dong Y., Li Q.X. (2021). Isoorientin, a GSK-3beta inhibitor, rescues synaptic dysfunction, spatial memory deficits and attenuates pathological progression in APP/PS1 model mice. Behav. Brain Res..

[78] Habtemariam S. (2018). Molecular pharmacology of rosmarinic and salvianolic acids: Potential seeds for Alzheimer’s and vascular dementia drugs. Int. J. Mol. Sci..

[79] Cornejo A., Aguilar Sandoval F., Caballero L., Machuca L., Munoz P., Caballero J., Perry G., Ardiles A., Areche C., Melo F. (2017). Rosmarinic acid prevents fibrillization and diminishes vibrational modes associated to beta sheet in tau protein linked to Alzheimer’s disease. J. Enzyme Inhib. Med. Chem..

[80] Taguchi R., Hatayama K., Takahashi T., Hayashi T., Sato Y., Sato D., Ohta K., Nakano H., Seki C., Endo Y. (2017). Structure-activity relations of rosmarinic acid derivatives for the amyloid beta aggregation inhibition and antioxidant properties. Eur. J. Med. Chem..

[81] Sun X., Zhu J., Sun X.Y., Ji M., Yu X.L., Liu R.T. (2020). Ellagic acid rescues motor and cognitive deficits in the R6/2 mouse model of Huntington’s disease by lowering mutant huntingtin protein. Food Funct..

[82] Xiao S., Lu Y., Wu Q., Yang J., Chen J., Zhong S., Eliezer D., Tan Q., Wu C. (2021). Fisetin inhibits tau aggregation by interacting with the protein and preventing the formation of beta-strands. Int. J. Biol. Macromol..

[83] Georgiadou G., Tarantilis P.A., Pitsikas N. (2012). Effects of the active constituents of *Crocus sativus* L. crocins, in an animal model of obsessive-compulsive disorder. Neurosci. Lett..

[84] Ardah M.T., Paleologou K.E., Lv G., Menon S.A., Abul Khair S.B., Lu J.H., Safieh-Garabedian B., Al-Hayani A.A., Eliezer D., Li M. (2015). Ginsenoside Rb1 inhibits fibrillation and toxicity of alpha-synuclein and disaggregates preformed fibrils. Neurobiol. Dis..

[85] Liu Y., Carver J.A., Calabrese A.N., Pukala T.L. (2014). Gallic acid interacts with alpha-synuclein to prevent the structural collapse necessary for its aggregation. Biochim. Biophys. Acta..

[86] Ardah M.T., Paleologou K.E., Lv G., Abul Khair S.B., Kazim A.S., Minhas S.T., Al-Tel T.H., Al-Hayani A.A., Haque M.E., Eliezer D. (2014). Structure activity relationship of phenolic acid inhibitors of alpha-synuclein fibril formation and toxicity. Front. Aging. Neurosci..

[87] D’Onofrio G., Sancarlo D., Ruan Q., Yu Z., Panza F., Daniele A., Greco A., Seripa D. (2017). Phytochemicals in the Treatment of Alzheimer’s Disease: A Systematic Review. Curr. Drug Targets.

[88] Thinakaran G., Koo E.H. (2008). Amyloid precursor protein trafficking, processing, and function. J. Biol. Chem..

[89] Ono K., Watanabe-Nakayama T. (2021). Aggregation and structure of amyloid β-protein. Neurochem. Int..

[90] Calfio C., Gonzalez A., Singh S.K., Rojo L.E., Maccioni R.B. (2020). The emerging Role of nutraceuticals and phytochemicals in the prevention and treatment of Alzheimer’s disease. J. Alzheimers Dis..

[91] Simunkova M., Alwasel S.H., Alhazza I.M., Jomova K., Kollar V., Rusko M., Valko M. (2019). Management of oxidative stress and other pathologies in Alzheimer’s disease. Arch. Toxicol..

[92] Peng Y., Ao M., Dong B., Jiang Y., Yu L., Chen Z., Hu C., Xu R. (2021). Anti-inflammatory effects of curcumin in the inflammatory diseases: Status, limitations and countermeasures. Drug Des. Dev. Ther..

[93] Ly P.T., Wu Y., Zou H., Wang R., Zhou W., Kinoshita A., Zhang M., Yang Y., Cai F., Woodgett J. (2013). Inhibition of GSK3β-mediated BACE1 expression reduces Alzheimer-associated phenotypes. J. Clin. Investig..

[94] Huang P., Zheng N., Zhou H.B., Huang J. (2020). Curcumin inhibits BACE1 expression through the interaction between ERbeta and NFkappaB signaling pathway in SH-SY5Y cells. Mol. Cell. Biochem..

[95] Wan Y., Liang Y., Liang F., Shen N., Shinozuka K., Yu J.T., Ran C., Quan Q., Tanzi R.E., Zhang C. (2019). A Curcumin analog reduces levels of the Alzheimer’s disease-associated amyloid-beta protein by modulating abetaPP processing and autophagy. J. Alzheimers Dis..

[96] Jakubowski J.M., Orr A.A., Le D.A., Tamamis P. (2020). Interactions between curcumin derivatives and amyloid-beta fibrils: Insights from molecular dynamics simulations. J. Chem. Inf. Model..

[97] Fidelis E.M., Savall A.S.P., da Luz Abreu E., Carvalho F., Teixeira F.E.G., Haas S.E., Bazanella Sampaio T., Pinton S. (2019). Curcumin-loaded nanocapsules reverses the depressant-like behavior and oxidative stress induced by beta-amyloid in mice. Neuroscience.

[98] Ruan Y., Xiong Y., Fang W., Yu Q., Mai Y., Cao Z., Wang K., Lei M., Xu J., Liu Y. (2022). Highly sensitive Curcumin-conjugated nanotheranostic platform for detecting amyloid-beta plaques by magnetic resonance imaging and reversing cognitive deficits of Alzheimer’s disease via NLRP3-inhibition. J. Nanobiotechnol..

[99] Mathew A., Fukuda T., Nagaoka Y., Hasumura T., Morimoto H., Yoshida Y., Maekawa T., Venugopal K., Kumar D.S. (2012). Curcumin loaded-PLGA nanoparticles conjugated with Tet-1 peptide for potential use in Alzheimer’s disease. PLoS ONE.

[100] Wang N., Zhou Y., Zhao L., Wang C., Ma W., Ge G., Wang Y., Ullah I., Muhammad F., Alwayli D. (2020). Ferulic acid delayed amyloid beta-induced pathological symptoms by autophagy pathway via a fasting-like effect in *Caenorhabditis elegans*. Food Chem. Toxicol..

[101] Wang N.Y., Li J.N., Liu W.L., Huang Q., Li W.X., Tan Y.H., Liu F., Song Z.H., Wang M.Y., Xie N. (2021). Ferulic acid ameliorates Alzheimer’s disease-like pathology and repairs cognitive decline by preventing capillary hypofunction in APP/PS1 Mice. Neurotherapeutics.

[102] Kim J.H., Wang Q., Choi J.M., Lee S., Cho E.J. (2015). Protective role of caffeic acid in an Aβ25-35-induced Alzheimer’s disease model. Nut. Res. Pract..

[103] Li H., Yu X., Li C., Ma L., Zhao Z., Guan S., Wang L. (2021). Caffeic acid protects against Abeta toxicity and prolongs lifespan in *Caenorhabditis elegans* models. Food Funct..

[104] Andrade S., Pereira M.C., Loureiro J.A. (2023). Caffeic acid loaded into engineered lipid nanoparticles for Alzheimer’s disease therapy. Colloids Surf. B Biointerfaces.

[105] Shimmyo Y., Kihara T., Akaike A., Niidome T., Sugimoto H. (2008). Flavonols and flavones as BACE-1 inhibitors: Structure-activity relationship in cell-free, cell-based and in silico studies reveal novel pharmacophore features. Biochim. Biophys. Acta.

[106] Paris D., Mathura V., Ait-Ghezala G., Beaulieu-Abdelahad D., Patel N., Bachmeier C., Mullan M. (2011). Flavonoids lower Alzheimer's Aβ production via an NFκB dependent mechanism. Bioinformation..

[107] Kong Y., Li K., Fu T., Wan C., Zhang D., Song H., Zhang Y., Liu N., Gan Z., Yuan L. (2016). Quercetin ameliorates Aβ toxicity in Drosophila AD model by modulating cell cycle-related protein expression. Oncotarget..

[108] Li Y., Zhou S., Li J., Sun Y., Hasimu H., Liu R., Zhang T. (2015). Quercetin protects human brain microvascular endothelial cells from fibrillar beta-amyloid1-40-induced toxicity. Acta. Pharm. Sin. B.

[109] Dong X., Tang Y., Zhan C., Wei G. (2021). Green tea extract EGCG plays a dual role in abeta(42) protofibril disruption and membrane protection: A molecular dynamic study. Chem. Phys. Lipids.

[110] Zhang J.S., Zhou S.F., Wang Q., Guo J.N., Liang H.M., Deng J.B., He W.Y. (2016). Gastrodin suppresses BACE1 expression under oxidative stress condition via inhibition of the PKR/eIF2alpha pathway in Alzheimer’s disease. Neuroscience.

[111] Li M., Qian S. (2016). Gastrodin protects neural progenitor cells against amyloid beta (1-42)-induced neurotoxicity and improves hippocampal neurogenesis in amyloid beta (1-42)-injected mice. J. Mol. Neurosci..

[112] Hu Y., Li C., Shen W. (2014). Gastrodin alleviates memory deficits and reduces neuropathology in a mouse model of Alzheimer’s disease. Neuropathology.

[113] Zhao X., Zou Y., Xu H., Fan L., Guo H., Li X., Li G., Zhang X., Dong M. (2012). Gastrodin protect primary cultured rat hippocampal neurons against amyloid-beta peptide-induced neurotoxicity via ERK1/2-Nrf2 pathway. Brain Res..

[114] Cao Y.Y., Wang L., Ge H., Lu X.L., Pei Z., Gu Q., Xu J. (2013). Salvianolic acid A, a polyphenolic derivative from Salvia miltiorrhiza bunge, as a multifunctional agent for the treatment of Alzheimer’s disease. Mol. Divers..

[115] Durairajan S.S., Yuan Q., Xie L., Chan W.S., Kum W.F., Koo I., Liu C., Song Y., Huang J.D., Klein W.L. (2008). Salvianolic acid B inhibits abeta fibril formation and disaggregates preformed fibrils and protects against abeta-induced cytotoxicty. Neuroche. Int..

[116] Lin Y.H., Liu A.H., Wu H.L., Westenbroek C., Song Q.L., Yu H.M., Ter Horst G.J., Li X.J. (2006). Salvianolic acid B, an antioxidant from salvia miltiorrhiza, prevents abeta(25-35)-induced reduction in BPRP in PC12 cells. Biochem. Biophys. Res. Commun..

[117] He Y., Jia K., Li L., Wang Q., Zhang S., Du J., Du H. (2018). Salvianolic acid B attenuates mitochondrial stress against abeta toxicity in primary cultured mouse neurons. Biochem. Biophys. Res. Commun..

[118] Yu T., Paudel P., Seong S.H., Kim J.A., Jung H.A., Choi J.S. (2018). Computational insights into beta-site amyloid precursor protein enzyme 1 (BACE1) inhibition by tanshinones and salvianolic acids from Salvia miltiorrhiza via molecular docking simulations. Comput. Biol. Chem..

[119] Baek S., Park S., Shin J., Lee J.-S., Kim H.Y., Han G., Kim Y. (2020). Investigation of memory-enhancing botanical mixture and their isolated compounds for inhibition of amyloid-β and tau aggregation. Appl. Biol. Chem..

[120] Jayatunga D.P.W., Hone E., Fernando W., Garg M.L., Verdile G., Martins R.N. (2021). Mitoprotective effects of a synergistic nutraceutical combination: Basis for a prevention strategy against Alzheimer’s disease. Front. Aging Neurosci..

[121] Dourado N.S., Souza C.D.S., de Almeida M.M.A., Bispo da Silva A., Dos Santos B.L., Silva V.D.A., De Assis A.M., da Silva J.S., Souza D.O., Costa M.F.D. (2020). Neuroimmunomodulatory and neuroprotective effects of the flavonoid apigenin in in vitro models of neuroinflammation associated with Alzheimer’s disease. Front. Aging Neurosci..

[122] Nikbakht F., Khadem Y., Haghani S., Hoseininia H., Moein Sadat A., Heshemi P., Jamali N. (2019). Protective role of apigenin against abeta 25-35 toxicity via inhibition of mitochondrial cytochrome crelease. Basic Clin. Neurosci..

[123] Siddique Y.H., Rahul, Ara G., Afzal M., Varshney H., Gaur K., Subhan I., Mantasha I., Shahid M. (2022). Beneficial effects of apigenin on the transgenic *Drosophila* model of Alzheimer’s disease. Chem. Biol. Interact..

[124] Min Z., Tang Y., Hu X.T., Zhu B.L., Ma Y.L., Zha J.S., Deng X.J., Yan Z., Chen G.J. (2018). Cosmosiin increases ADAM10 expression via mechanisms involving 5’UTR and PI3K signaling. Front. Mol. Neurosci..

[125] Gu L., Cai N., Li M., Bi D., Yao L., Fang W., Wu Y., Hu Z., Liu Q., Lin Z. (2022). Inhibitory effects of macelignan on tau phosphorylation and abeta aggregation in the cell model of Alzheimer’s disease. Front. Nutr..

[126] Hitl M., Kladar N., Gavarić N., Božin B. (2021). Rosmarinic acid-human pharmacokinetics and health benefits. Planta Medica.

[127] Lee A.Y., Hwang B.R., Lee M.H., Lee S., Cho E.J. (2016). Perilla frutescens var. japonica and rosmarinic acid improve amyloid-beta25-35 induced impairment of cognition and memory function. Nutr. Res. Pract..

[128] Mirza F.J., Amber S., Sumera, Hassan D., Ahmed T., Zahid S. (2021). Rosmarinic acid and ursolic acid alleviate deficits in cognition, synaptic regulation and adult hippocampal neurogenesis in an abeta(1-42)-induced mouse model of Alzheimer’s disease. Phytomedicine.

[129] Dani M., Brooks D.J., Edison P. (2016). Tau imaging in neurodegenerative diseases. Eur. J. Nucl. Med. Mol. Imaging.

[130] Giacobini E., Gold G. (2013). Alzheimer disease therapy--moving from amyloid-β to tau. Nat. Rev. Neurol..

[131] Spillantini M.G., Goedert M. (2013). Tau pathology and neurodegeneration. Lancet Neurol..

[132] Lee H.G., Perry G., Moreira P.I., Garrett M.R., Liu Q., Zhu X., Takeda A., Nunomura A., Smith M.A. (2005). Tau phosphorylation in Alzheimer’s disease: Pathogen or protector?. Trends Mol. Med..

[133] Chabrier M.A., Cheng D., Castello N.A., Green K.N., LaFerla F.M. (2014). Synergistic effects of amyloid-beta and wild-type human tau on dendritic spine loss in a floxed double transgenic model of Alzheimer’s disease. Neurobiol. Dis..

[134] Cleveland D.W., Hwo S.Y., Kirschner M.W. (1977). Physical and chemical properties of purified tau factor and the role of tau in microtubule assembly. J. Mol. Bio..

[135] Ballatore C., Lee V.M., Trojanowski J.Q. (2007). Tau-mediated neurodegeneration in Alzheimer’s disease and related disorders. Nat. Rev. Neurosci..

[136] Herrup K., Carrillo M.C., Schenk D., Cacace A., Desanti S., Fremeau R., Bhat R., Glicksman M., May P., Swerdlow R. (2013). Beyond amyloid: Getting real about nonamyloid targets in Alzheimer’s disease. Alzheimers Dement..

[137] Stoothoff W.H., Johnson G.V. (2005). Tau phosphorylation: Physiological and pathological consequences. Biochim. Biophys. Acta.

[138] von Bergen M., Barghorn S., Biernat J., Mandelkow E.M., Mandelkow E. (2005). Tau aggregation is driven by a transition from random coil to beta sheet structure. Biochim. Biophys. Acta.

[139] Mietelska-Porowska A., Wasik U., Goras M., Filipek A., Niewiadomska G. (2014). Tau protein modifications and interactions: Their role in function and dysfunction. Int. J. Mol. Sci..

[140] Goedert M., Ghetti B., Spillantini M.G. (2000). Tau gene mutations in frontotemporal dementia and parkinsonism linked to chromosome 17 (FTDP-17). Their relevance for understanding the neurogenerative process. Ann. N. Y. Acad. Sci..

[141] Hutton M. (2000). Molecular genetics of chromosome 17 tauopathies. Ann. N. Y. Acad. Sci..

[142] Shafiei S.S., Guerrero-Munoz M.J., Castillo-Carranza D.L. (2017). Tau oligomers: Cytotoxicity, propagation, and mitochondrial damage. Front. Aging Neurosci..

[143] Congdon E.E., Sigurdsson E.M. (2018). Tau-targeting therapies for Alzheimer disease. Nat. Rev. Neurol..

[144] Kim S., Choi K.J., Cho S.J., Yun S.M., Jeon J.P., Koh Y.H., Song J., Johnson G.V., Jo C. (2016). Fisetin stimulates autophagic degradation of phosphorylated tau via the activation of TFEB and Nrf2 transcription factors. Sci. Rep..

[145] Ait-Bouziad N., Lv G., Mahul-Mellier A.L., Xiao S., Zorludemir G., Eliezer D., Walz T., Lashuel H.A. (2017). Discovery and characterization of stable and toxic Tau/phospholipid oligomeric complexes. Nat. Commun..

[146] Ahmad A., Ali T., Park H.Y., Badshah H., Rehman S.U., Kim M.O. (2017). Neuroprotective effect of fisetin against amyloid-beta-induced cognitive/synaptic dysfunction, neuroinflammation, and neurodegeneration in adult mice. Mol. Neurobiol..

[147] Soeda S., Ochiai T., Paopong L., Tanaka H., Shoyama Y., Shimeno H. (2001). Crocin suppresses tumor necrosis factor-alpha-induced cell death of neuronally differentiated PC-12 cells. Life Sci..

[148] Ochiai T., Ohno S., Soeda S., Tanaka H., Shoyama Y., Shimeno H. (2004). Crocin prevents the death of rat pheochromyctoma (PC-12) cells by its antioxidant effects stronger than those of alpha-tocopherol. Neurosci. Lett..

[149] Inoue E., Shimizu Y., Masui R., Hayakawa T., Tsubonoya T., Hori S., Sudoh K. (2018). Effects of saffron and its constituents, crocin-1, crocin-2, and crocetin on alpha-synuclein fibrils. J. Nat. Med..

[150] Sugiura M., Shoyama Y., Saito H., Abe K. (1995). The effects of ethanol and crocin on the induction of long-term potentiation in the CA1 region of rat hippocampal slices. Jap. J. Of. Pharmacol..

[151] Karakani A.M., Riazi G., Ghaffari S.M., Ahmadian S., Mokhtari F., Firuzi M.J., Bathaie S.Z. (2015). Inhibitory effect of corcin on aggregation of 1N/4R human tau protein in vitro. Iran. J. Basic Med. Sci..

[152] Rashedinia M., Lari P., Abnous K., Hosseinzadeh H. (2015). Protective effect of crocin on acrolein-induced tau phosphorylation in the rat brain. Acta Neurobiol. Exp..

[153] Chalatsa I., Arvanitis D.A., Koulakiotis N.S., Giagini A., Skaltsounis A.L., Papadopoulou-Daifoti Z., Tsarbopoulos A., Sanoudou D. (2019). The crocus sativus compounds trans-crocin 4 and trans-crocetin modulate the amyloidogenic pathway and tau misprocessing in Alzheimer disease neuronal cell culture models. Front. Neurosci..

[154] Burns J., Yokota T., Ashihara H., Lean M.E., Crozier A. (2002). Plant foods and herbal sources of resveratrol. J. Agric. Food. Chem..

[155] Baur J.A., Sinclair D.A. (2006). Therapeutic potential of resveratrol: The in vivo evidence. Nat. Rev. Drug Discov..

[156] Jeon B.T., Jeong E.A., Shin H.J., Lee Y., Lee D.H., Kim H.J., Kang S.S., Cho G.J., Choi W.S., Roh G.S. (2012). Resveratrol attenuates obesity-associated peripheral and central inflammation and improves memory deficit in mice fed a high-fat diet. Diabetes.

[157] Gong C.X., Shaikh S., Wang J.Z., Zaidi T., Grundke-Iqbal I., Iqbal K. (1995). Phosphatase activity toward abnormally phosphorylated tau: Decrease in Alzheimer disease brain. J. Neurochem..

[158] Gong C.X., Singh T.J., Grundke-Iqbal I., Iqbal K. (1993). Phosphoprotein phosphatase activities in Alzheimer disease brain. J. Neurochem..

[159] Vogelsberg-Ragaglia V., Schuck T., Trojanowski J.Q., Lee V.M. (2001). PP2A mRNA expression is quantitatively decreased in Alzheimer’s disease hippocampus. Exp. Neurol..

[160] Wang Y.J., Chen G.H., Hu X.Y., Lu Y.P., Zhou J.N., Liu R.Y. (2005). The expression of calcium/calmodulin-dependent protein kinase II-alpha in the hippocampus of patients with Alzheimer’s disease and its links with AD-related pathology. Brain Res..

[161] Pei J.J., Braak E., Braak H., Grundke-Iqbal I., Iqbal K., Winblad B., Cowburn R.F. (1999). Distribution of active glycogen synthase kinase 3beta (GSK-3beta) in brains staged for Alzheimer disease neurofibrillary changes. J. Neurotaph. Exp. Neur..

[162] He X., Li Z., Rizak J.D., Wu S., Wang Z., He R., Su M., Qin D., Wang J., Hu X. (2016). Resveratrol attenuates formaldehyde induced hyperphosphorylation of tau protein and cytotoxicity in N2a cells. Front. Neurosci..

[163] Trockenbacher A., Suckow V., Foerster J., Winter J., Krauss S., Ropers H.H., Schneider R., Schweiger S. (2001). MID1, mutated in Opitz syndrome, encodes an ubiquitin ligase that targets phosphatase 2A for degradation. Nat. Genet..

[164] Schweiger S., Matthes F., Posey K., Kickstein E., Weber S., Hettich M.M., Pfurtscheller S., Ehninger D., Schneider R., Krauß S. (2017). Resveratrol induces dephosphorylation of Tau by interfering with the MID1-PP2A complex. Sci. Rep..

[165] Shati A.A., Alfaifi M.Y. (2019). Trans-resveratrol Inhibits Tau phosphorylation in the brains of control and cadmium chloride-treated rats by activating PP2A and PI3K/Akt induced-inhibition of GSK3beta. Neurochem. Res..

[166] Jhang K.A., Park J.S., Kim H.S., Chong Y.H. (2017). Resveratrol ameliorates tau hyperphosphorylation at Ser396 Site and oxidative damage in rat hippocampal slices exposed to vanadate: Implication of ERK1/2 and GSK-3beta signaling cascades. J. Agric. Food Chem..

[167] Means J.C., Lopez A.A., Koulen P. (2020). Resveratrol protects optic nerve head astrocytes from oxidative stress-induced cell death by preventing caspase-3 activation, tau dephosphorylation at Ser(422) and formation of misfolded protein aggregates. Cell. Mol. Neurobiol..

[168] Ishisaka A., Mukai R., Terao J., Shibata N., Kawai Y. (2014). Specific localization of quercetin-3-O-glucuronide in human brain. Arch. Biochem. Biophys..

[169] Chen J., Deng X., Liu N., Li M., Liu B., Fu Q., Qu R., Ma S. (2016). Quercetin attenuates tau hyperphosphorylation and improves cognitive disorder via suppression of ER stress in a manner dependent on AMPK pathway. J. Funct. Foods.

[170] Kimura T., Ishiguro K., Hisanaga S. (2014). Physiological and pathological phosphorylation of tau by Cdk5. Front. Mol. Neurosci..

[171] Shen X.Y., Luo T., Li S., Ting O.Y., He F., Xu J., Wang H.Q. (2018). Quercetin inhibits okadaic acid-induced tau protein hyperphosphorylation through the Ca2+-calpain-p25-CDK5 pathway in HT22 cells. Int. J. Mol. Med..

[172] Huang H.C., Xu K., Jiang Z.F. (2012). Curcumin-mediated neuroprotection against amyloid-beta-induced mitochondrial dysfunction involves the inhibition of GSK-3beta. J. Alzheimers Dis..

[173] Huang H.C., Tang D., Xu K., Jiang Z.F. (2014). Curcumin attenuates amyloid-beta-induced tau hyperphosphorylation in human neuroblastoma SH-SY5Y cells involving PTEN/Akt/GSK-3beta signaling pathway. J. Recept. Signal Transduct. Res..

[174] Wobst H.J., Sharma A., Diamond M.I., Wanker E.E., Bieschke J. (2015). The green tea polyphenol (-)-epigallocatechin gallate prevents the aggregation of tau protein into toxic oligomers at substoichiometric ratios. FEBS Lett..

[175] Chesser A.S., Ganeshan V., Yang J., Johnson G.V. (2016). Epigallocatechin-3-gallate enhances clearance of phosphorylated tau in primary neurons. Nutr. Neurosci..

[176] Gong E.J., Park H.R., Kim M.E., Piao S., Lee E., Jo D.G., Chung H.Y., Ha N.C., Mattson M.P., Lee J. (2011). Morin attenuates tau hyperphosphorylation by inhibiting GSK3beta. Neurobiol. Dis..

[177] Du Y., Qu J., Zhang W., Bai M., Zhou Q., Zhang Z., Li Z., Miao J. (2016). Morin reverses neuropathological and cognitive impairments in APPswe/PS1dE9 mice by targeting multiple pathogenic mechanisms. Neuropharmacology.

[178] An F., Bai Y., Xuan X., Bian M., Zhang G., Wei C. (2022). 1,8-Cineole ameliorates advanced glycation end products-induced Alzheimer’s disease-like pathology in vitro and in vivo. Molecules.

[179] Nakajima A., Aoyama Y., Nguyen T.T., Shin E.J., Kim H.C., Yamada S., Nakai T., Nagai T., Yokosuka A., Mimaki Y. (2013). Nobiletin, a citrus flavonoid, ameliorates cognitive impairment, oxidative burden, and hyperphosphorylation of tau in senescence-accelerated mouse. Behav. Brain Res..

[180] Huang X.T., Qian Z.M., He X., Gong Q., Wu K.C., Jiang L.R., Lu L.N., Zhu Z.J., Zhang H.Y., Yung W.H. (2014). Reducing iron in the brain: A novel pharmacologic mechanism of huperzine A in the treatment of Alzheimer’s disease. Neurobiol. Aging.

[181] Laurent C., Eddarkaoui S., Derisbourg M., Leboucher A., Demeyer D., Carrier S., Schneider M., Hamdane M., Muller C.E., Buee L. (2014). Beneficial effects of caffeine in a transgenic model of Alzheimer’s disease-like tau pathology. Neurobiol. Aging.

[182] Shasaltaneh M.D., Naghdi N., Ramezani S., Alizadeh L., Riazi G.H. (2022). Protection of beta boswellic acid against streptozotocin-induced Alzheimer’s model by reduction of tau phosphorylation level and enhancement of reelin expression. Planta Med..

[183] Xiao S., Wu Q., Yao X., Zhang J., Zhong W., Zhao J., Liu Q., Zhang M. (2021). Inhibitory effects of isobavachalcone on tau protein aggregation, tau phosphorylation, and oligomeric tau-induced apoptosis. ACS Chem. Neurosci..

[184] Gorantla N.V., Das R., Mulani F.A., Thulasiram H.V., Chinnathambi S. (2019). Neem derivatives inhibits tau aggregation. J. Alzheimers Dis. Rep..

[185] Khare S.D., Chinchilla P., Baum J. (2023). Multifaceted interactions mediated by intrinsically disordered regions play key roles in alpha synuclein aggregation. Curr. Opin. Struc. Biol..

[186] Murphy D.D., Rueter S.M., Trojanowski J.Q., Lee V.M. (2000). Synucleins are developmentally expressed, and alpha-synuclein regulates the size of the presynaptic vesicular pool in primary hippocampal neurons. J. Neurosci..

[187] Uversky V.N., Lee H.J., Li J., Fink A.L., Lee S.J. (2001). Stabilization of partially folded conformation during alpha-synuclein oligomerization in both purified and cytosolic preparations. J. Biol. Chem..

[188] Paillusson S., Gomez-Suaga P., Stoica R., Little D., Gissen P., Devine M.J., Noble W., Hanger D.P., Miller C.C.J. (2017). α-Synuclein binds to the ER-mitochondria tethering protein VAPB to disrupt Ca(2+) homeostasis and mitochondrial ATP production. Acta Neuropathol..

[189] Lindström V., Gustafsson G., Sanders L.H., Howlett E.H., Sigvardson J., Kasrayan A., Ingelsson M., Bergström J., Erlandsson A. (2017). Extensive uptake of α-synuclein oligomers in astrocytes results in sustained intracellular deposits and mitochondrial damage. Mol. Cell. Neurosci..

[190] Uversky V.N. (2015). Hot, Hotter, and Hottest Trends in α-Synuclein Research. Curr. Protein. Pept. Sci..

[191] Büeler H. (2009). Impaired mitochondrial dynamics and function in the pathogenesis of Parkinson’s disease. Exp. Neurol..

[192] Niranjan R. (2014). The role of inflammatory and oxidative stress mechanisms in the pathogenesis of Parkinson’s disease: Focus on astrocytes. Mol. Neurobiol..

[193] Li J., Zhu M., Rajamani S., Uversky V.N., Fink A.L. (2004). Rifampicin inhibits alpha-synuclein fibrillation and disaggregates fibrils. Chem. Biol..

[194] Goel A., Kunnumakkara A.B., Aggarwal B.B. (2008). Curcumin as “Curecumin”: From kitchen to clinic. Biochem. Pharmacol..

[195] Cole G.M., Teter B., Frautschy S.A. (2007). Neuroprotective effects of curcumin. Ad. Exp. Med. Biol..

[196] Lim G.P., Chu T., Yang F., Beech W., Frautschy S.A., Cole G.M. (2001). The curry spice curcumin reduces oxidative damage and amyloid pathology in an Alzheimer transgenic mouse. J. Neurosci..

[197] Yang F., Lim G.P., Begum A.N., Ubeda O.J., Simmons M.R., Ambegaokar S.S., Chen P.P., Kayed R., Glabe C.G., Frautschy S.A. (2005). Curcumin inhibits formation of amyloid beta oligomers and fibrils, binds plaques, and reduces amyloid in vivo. J. Biol. Chem..

[198] Sharma N., Nehru B. (2018). Curcumin affords neuroprotection and inhibits alpha-synuclein aggregation in lipopolysaccharide-induced Parkinson’s disease model. Inflammopharmacology.

[199] Ahsan N., Mishra S., Jain M.K., Surolia A., Gupta S. (2015). Curcumin Pyrazole and its derivative (N-(3-Nitrophenylpyrazole) Curcumin inhibit aggregation, disrupt fibrils and modulate toxicity of Wild type and Mutant alpha-Synuclein. Sci. Rep..

[200] Singh B.N., Shankar S., Srivastava R.K. (2011). Green tea catechin, epigallocatechin-3-gallate (EGCG): Mechanisms, perspectives and clinical applications. Biochem. Pharmacol..

[201] Masuda M., Suzuki N., Taniguchi S., Oikawa T., Nonaka T., Iwatsubo T., Hisanaga S., Goedert M., Hasegawa M. (2006). Small molecule inhibitors of alpha-synuclein filament assembly. Biochemistry.

[202] Lorenzen N., Nielsen S.B., Yoshimura Y., Vad B.S., Andersen C.B., Betzer C., Kaspersen J.D., Christiansen G., Pedersen J.S., Jensen P.H. (2014). How epigallocatechin gallate can inhibit α-synuclein oligomer toxicity in vitro. J. Biol. Chem..

[203] Lee J.H., Hong C.S., Lee S., Yang J.E., Park Y.I., Lee D., Hyeon T., Jung S., Paik S.R. (2012). Radiating amyloid fibril formation on the surface of lipid membranes through unit-assembly of oligomeric species of α-synuclein. PLoS ONE.

[204] Yang J.E., Rhoo K.Y., Lee S., Lee J.T., Park J.H., Bhak G., Paik S.R. (2017). EGCG-mediated protection of the membrane disruption and cytotoxicity caused by the ‘active oligomer’ of alpha-synuclein. Sci. Rep..

[205] Liu X., Zhou S., Shi D., Bai Q., Liu H., Yao X. (2018). Influence of EGCG on alpha-synuclein (alphaS) aggregation and identification of their possible binding mode: A computational study using molecular dynamics simulation. Chem. Biol. Drug Des..

[206] Yao Y., Tang Y., Wei G. (2020). Epigallocatechin Gallate Destabilizes alpha-Synuclein Fibril by Disrupting the E46-K80 Salt-Bridge and Inter-protofibril Interface. ACS Chem. Neurosci..

[207] Dehay B., Martinez-Vicente M., Caldwell G.A., Caldwell K.A., Yue Z., Cookson M.R., Klein C., Vila M., Bezard E. (2013). Lysosomal impairment in Parkinson's disease. Movement. Disorders..

[208] Bourdenx M., Bezard E., Dehay B. (2014). Lysosomes and α-synuclein form a dangerous duet leading to neuronal cell death. Front. Neuroanat..

[209] Gu W.Z., Chen R., Brandwein S., McAlpine J., Burres N. (1995). Isolation, purification, and characterization of immunosuppressive compounds from tripterygium: Triptolide and tripdiolide. Int. J. Immunopharmaco..

[210] Zheng Y., Zhang W.J., Wang X.M. (2013). Triptolide with potential medicinal value for diseases of the central nervous system. CNS Neurosci. Ther..

[211] Hu G., Gong X., Wang L., Liu M., Liu Y., Fu X., Wang W., Zhang T., Wang X. (2017). Triptolide promotes the clearance of alpha-synuclein by enhancing autophagy in neuronal cells. Mol. Neurobiol..

[212] Eskandari H., Ghanadian M., Noleto-Dias C., Lomax C., Tawfike A., Christiansen G., Sutherland D.S., Ward J.L., Mohammad-Beigi H., Otzen D.E. (2020). Inhibitors of alpha-synuclein fibrillation and oligomer toxicity in rosa damascena: The all-pervading powers of flavonoids and phenolic glycosides. ACS Chem. Neurosci..

[213] Gallardo-Fernandez M., Hornedo-Ortega R., Cerezo A.B., Troncoso A.M., Garcia-Parrilla M.C. (2019). Melatonin, protocatechuic acid and hydroxytyrosol effects on vitagenes system against alpha-synuclein toxicity. Food. Chem. Toxicol..

[214] Tabrizi S.J., Ghosh R., Leavitt B.R. (2019). Huntingtin lowering strategies for disease modification in Huntington’s disease. Neuron.

[215] Caron N.S., Wright G.E.B., Hayden M.R., Adam M.P., Mirzaa G.M., Pagon R.A., Wallace S.E., Bean L.J.H., Gripp K.W., Amemiya A. (1993–2023). Huntington Disease. GeneReviews.

[216] Gil J.M., Rego A.C. (2008). Mechanisms of neurodegeneration in Huntington’s disease. Eur. J. Neurosci..

[217] Franco-Iborra S., Plaza-Zabala A., Montpeyo M., Sebastian D., Vila M., Martinez-Vicente M. (2021). Mutant HTT (huntingtin) impairs mitophagy in a cellular model of Huntington disease. Autophagy.

[218] Landrum E., Wetzel R. (2014). Biophysical underpinnings of the repeat length dependence of polyglutamine amyloid formation. J. Biol. Chem..

[219] Legleiter J., Mitchell E., Lotz G.P., Sapp E., Ng C., DiFiglia M., Thompson L.M., Muchowski P.J. (2010). Mutant huntingtin fragments form oligomers in a polyglutamine length-dependent manner in vitro and in vivo. J. Biol. Chem..

[220] Lum P.T., Sekar M., Gan S.H., Bonam S.R., Shaikh M.F. (2021). Protective effect of natural products against Huntington’s disease: An overview of scientific evidence and understanding their mechanism of action. ACS Chem. Neurosci..

[221] Kim A., Lalonde K., Truesdell A., Gomes Welter P., Brocardo P.S., Rosenstock T.R., Gil-Mohapel J. (2021). New avenues for the treatment of Huntington’s disease. Int. J. Mol. Sci..

[222] Gupta M., Sanjana, Singh N., Singh B., Alam P. (2022). Role of natural products in alleviation of Huntington’s disease: An overview. S. Afr. J. Bot..

[223] Ehrnhoefer D.E., Duennwald M., Markovic P., Wacker J.L., Engemann S., Roark M., Legleiter J., Marsh J.L., Thompson L.M., Lindquist S. (2006). Green tea (-)-epigallocatechin-gallate modulates early events in huntingtin misfolding and reduces toxicity in Huntington’s disease models. Hum. Mol. Genet..

[224] Varga J., Der N.P., Zsindely N., Bodai L. (2020). Green tea infusion alleviates neurodegeneration induced by mutant Huntingtin in *Drosophila*. Nutr. Neurosci..

[225] Beasley M., Stonebraker A.R., Hasan I., Kapp K.L., Liang B.J., Agarwal G., Groover S., Sedighi F., Legleiter J. (2019). Lipid membranes influence the ability of small molecules to inhibit Huntingtin fibrillization. Biochemistry.

[226] Jain S., Panuganti V., Jha S., Roy I. (2020). Harmine acts as an indirect inhibitor of intracellular protein aggregation. ACS Omega.

[227] Portz B., Lee B.L., Shorter J. (2021). FUS and TDP-43 phases in health and disease. Trends Biochem. Sci..

[228] Ji Y., Li F., Qiao Y. (2022). Modulating liquid-liquid phase separation of FUS: Mechanisms and strategies. J. Mater. Chem. B.

[229] Dormann D., Haass C. (2013). Fused in sarcoma (FUS): An oncogene goes awry in neurodegeneration. Mol. Cell. Neurosci..

[230] Reber S., Jutzi D., Lindsay H., Devoy A., Mechtersheimer J., Levone B.R., Domanski M., Bentmann E., Dormann D., Mühlemann O. (2021). The phase separation-dependent FUS interactome reveals nuclear and cytoplasmic function of liquid-liquid phase separation. Nucleic Acids Res..

[231] Carey J.L., Guo L. (2022). Liquid-Liquid Phase Separation of TDP-43 and FUS in physiology and pathology of neurodegenerative diseases. Front. Mol. Biosci..

[232] Lenard A.J., Zhou Q., Madreiter-Sokolowski C., Bourgeois B., Habacher H., Khanna Y., Madl T. (2022). EGCG promotes FUS condensate formation in a methylation-dependent manner. Cells.

[233] Hu S., Maiti P., Ma Q., Zuo X., Jones M.R., Cole G.M., Frautschy S.A. (2015). Clinical development of curcumin in neurodegenerative disease. Expert. Rev. Neurother..

[234] Rigacci S., Stefani M. (2015). Nutraceuticals and amyloid neurodegenerative diseases: A focus on natural phenols. Expert. Rev. Neurother..

[235] Singh M., Arseneault M., Sanderson T., Murthy V., Ramassamy C. (2008). Challenges for research on polyphenols from foods in Alzheimer’s disease: Bioavailability, metabolism, and cellular and molecular mechanisms. J. Agr. Food Chem..

[236] Zhao D., Simon J.E., Wu Q. (2020). A critical review on grape polyphenols for neuroprotection: Strategies to enhance bioefficacy. Crit. Rev. Food Sci. Nutr..

[237] Bilia A.R., Piazzini V., Risaliti L., Vanti G., Casamonti M., Wang M., Bergonzi M.C. (2019). Nanocarriers: A successful tool to increase solubility, stability and optimise bioefficacy of natural constituents. Curr. Med. Chem..

[238] Renaud J., Martinoli M.G. (2019). Considerations for the use of polyphenols as therapies in neurodegenerative diseases. Int. J. Mol. Sci..

